# A Genome-Wide Identification of the WRKY Family Genes and a Survey of Potential WRKY Target Genes in *Dendrobium officinale*

**DOI:** 10.1038/s41598-017-07872-8

**Published:** 2017-08-23

**Authors:** Chunmei He, Jaime A. Teixeira da Silva, Jianwen Tan, Jianxia Zhang, Xiaoping Pan, Mingzhi Li, Jianping Luo, Jun Duan

**Affiliations:** 1 Key Laboratory of South China Agricultural Plant Molecular Analysis and Gene Improvement, South China Botanical Garden, Chinese Academy of Sciences, Guangzhou, 510650 China; 2 P. O. Box 7, Miki-cho post office, Ikenobe 3011-2, Miki-cho, Kita-gun Kagawa-ken, 761-0799 Japan; 3 College of Forestry and Landscape Architecture, South China Agricultural University, Guangzhou, 510642 China; 4 Genepioneer Biotechnologies Co. Ltd, Nanjing, 210014 China; 5 School of Food Science and Engineering, Hefei University of Technology, Hefei, 230009 China

## Abstract

The WRKY family, one of the largest families of transcription factors, plays important roles in the regulation of various biological processes, including growth, development and stress responses in plants. In the present study, 63 *DoWRKY* genes were identified from the *Dendrobium officinale* genome. These were classified into groups I, II, III and a non-group, each with 14, 28, 10 and 11 members, respectively. ABA-responsive, sulfur-responsive and low temperature-responsive elements were identified in the 1-k upstream regulatory region of *DoWRKY* genes. Subsequently, the expression of the 63 *DoWRKY* genes under cold stress was assessed, and the expression profiles of a large number of these genes were regulated by low temperature in roots and stems. To further understand the regulatory mechanism of *DoWRKY* genes in biological processes, potential WRKY target genes were investigated. Among them, most stress-related genes contained multiple W-box elements in their promoters. In addition, the genes involved in polysaccharide synthesis and hydrolysis contained W-box elements in their 1-k upstream regulatory regions, suggesting that *DoWRKY* genes may play a role in polysaccharide metabolism. These results provide a basis for investigating the function of *WRKY* genes and help to understand the downstream regulation network in plants within the *Orchidaceae*.

## Introduction

Transcription factors (TFs) are proteins that bind to specific DNA sequences and regulate the downstream expression of genes at the level of transcription, thereby influencing and controlling various biological processes^1^. Among the TF families, the WRKY family is a superfamily of TFs with 88 and 129 members in Arabidopsis (*Arabidopsis thalian*a) and rice (*Oryza sativa*), respectively (http://plntfdb.bio.uni-potsdam.de/v3.0/). WRKY proteins contain one or two highly conserved amino acid sequences, namely WRKY domain (WRKYGQK), with one or two zinc-finger-like motifs^2, 3^. The WRKY domain and zinc-finger-like motif have a DNA-binding domain that is responsible for the recognition of the W-box sequence, (C/T)TGAC(T/C)^2, 4^. Based on the number of WRKY domains and the type of zinc-finger motifs, WRKY proteins have been classified into three main groups: group I, II and III^2, 3, 5^. In addition, group II was subdivided into five subgroups, IIa, IIb, IIc, IId and IIe, based on phylogenetic analyses^3^. WRKY proteins in group I contain two WRKY domains and two zinc-finger motifs^2, 6^. Both group II and III WRKY proteins contain a single WRKY domain and a zinc-finger motif, while group III proteins have a zinc-finger motif with a C-C-H-C zinc-finger structure rather than C-C-H-H^2, 3, 5^.

The first *WRKY* gene (*SPF1*) from sweet potato (*Ipomoea batatas*) was identified and characterized in 1994^7^. Since then, numerous *WRKY* genes have been cloned and characterized from various plant species such as wheat (*Triticum aestivum*)^8^, soybean (*Glycine max*)^9^, rice^10^ and even an orchid, *Dendrobium officinale* ^11^. WRKY family members have also been identified and analyzed at the genome level. To date, genome-wide WRKY analyses have been performed in various plant species including arabidopsis (*Arabidopsis thaliana*)^2^, rice^6^, cucumber (*Cucumis sativus*)^12^, *Brachypodium distachyon* ^13^, birdsfoot trefoil (*Lotus japonicas*)^14^, grape^15^, carrot (*Daucus carota*)^16^, cassava (*Manihot esculenta*)^17^, and other plants.

Generally, WRKY proteins are regarded as positive or negative regulators and play a broad-spectrum regulatory role in developmental and physiological processes. In plants, WRKY proteins have been demonstrated to act in the growth of leaves and stems^18^, senescence^19^ and dormancy^20^. Accumulating data has also demonstrated that WRKY proteins play regulatory roles in biotic stress caused by viruses^21^, bacterial pathogens^22^, fungi^23^ and oomycetes^24^, as well as in various abiotic stresses, including wounding, cold, heat, drought or salinity^25^. The regulation of *WRKY* genes in abiotic stress has been increasingly characterized in recent years. For example, a WRKY TF *AtWRKY46* regulated osmotic stress responses and stomatal movement in *A. thaliana* ^26^. GmWRKY27 interacted with GmMYB174 to reduce the expression of a negative stress tolerance factor GmNAC29 to improve salt and drought tolerance^27^. Wheat *TaWRKY*2 and *TaWRKY44* genes are involved in multiple abiotic stress tolerance, including to drought, salt, freezing and osmotic stress^28, 29^.

*D*. *officinale* is an important traditional Chinese medicine^30^. Studies on TFs in *D*. *officinale*, or even in other orchids, are rarely reported, although genomic data for *D*. *officinale* and other orchids has emerged in the past two years^31–33^. In this study, a total of 63 *WRKY* genes from *D*. *officinale* were identified, analyzed or classified, and their conserved motif composition and expression were assessed under cold stress. Furthermore, potential WRKY target genes were investigated and annotated. Comprehensive studies of the WRKY family genes and WRKY target genes in *D*. *officinale* will shed light on the functions of this TF family in orchids.

## Materials and Methods

### Plant materials and stress treatments

*D*. *officinale* seedlings, which were used for the cold stress treatment, were cultured on half-strength Murashige and Skoog^34^ (MS) medium containing 2% sucrose and 0.6% agar (pH 5.4), in a growth chamber (26 ± 1 °C, 40 µmol m^−2^ s^−1^, a 12-h photoperiod and 60% relative humidity). To detect the expression of WRKY family genes under cold stress, plantlets about 10 months after germination and 8–9 cm in height were subjected to cold stress treatment. Plantlets grown on agar-based medium were carefully removed and transferred to half-strength MS liquid medium containing 2% sucrose (pH 5.4), and used as the control. For cold stress, plantlets on the same medium as the control were transferred to a 4 °C growth chamber. The roots and stems were harvested from four time points (0 h, 2 h, 6 h and 12 h), frozen in liquid nitrogen and stored at −70 °C within three days. Six plantlets were pooled as one biological replicate and for each experiment there were three biological replicates.

### Identification of *WRKY* genes in *D*. *officinale* and phylogenetic analysis

The Coding DNA Sequence (CDS) file of *D*. *officinale* was downloaded from the Herbal Medicine Omics Database (http://202.203.187.112/herbalplant/)^32^. The hidden Markov model (HMM) profile of WRKY with accession number PF03106 was downloaded from the Pfam database (http://pfam.xfam.org/). All putative DoWRKY TFs were obtained by screening *D*. *officinale* protein sequences using HMMER 3.0 software (http://hmmer.janelia.org/). The putative DoWRKY sequences were checked by the National Center for Biotechnology Information (NCBI) Basic Local Alignment Search Tool (BLAST). All putative DoWRKY proteins that were confirmed to be WRKY proteins in the NCBI database were considered as DoWRKY proteins. DoWRKY proteins without a WRKYGQK motif and redundant genes were discarded. The proteins containing the WRKYGQK domain without a zinc-finger structure were perceived as incomplete genes and 3′ ends were generated by a SMARTer RACE cDNA Amplification Kit (Clontech Laboratories; see supplementary method 1). All the remaining validated protein DoWRKY sequences and selected AtWRKY proteins (detailed information in Supplementary text 1) were aligned using ClustalX version 2.1^35^ and a phylogenetic tree was constructed with a bootstrapped Neighbor-Joining (NJ) method.

### Conserved motif distributions and gene structure analysis

Conserved motifs for each DoWRKY amino acid sequence were analyzed by Multiple Em for Motif Elicitation (MEME) Suite (version 4.11.2; http://meme.nbcr.net/meme/). The parameters for motif identification were set as follows: maximum number, 20; site distribution, any number of repetitions; minimum width, 10; and maximum width, 50. For gene structure analysis, the corresponding genome sequences of *DoWRKY* genes were obtained from the genome sequences of *D*. *officinale* which were downloaded from the Herbal Medicine Omics Database (http://202.203.187.112/herbalplant/)^32^ and from the whole genome sequence of *D*. *officinale* (DDBJ/EMBL/GenBank accession code: JSDN00000000)^33^. Genomic and CDS sequences were used for drawing gene structure schematic diagrams with the Gene Structure Display Server from the Center for Bioinformatics at Peking University (http://gsds.cbi.pku.edu.cn/index.php)^36^.

### Analysis of the *cis*-regulatory elements in the promoters of *DoWRKY* genes

The upstream 1-k (kilobase) regulatory regions (from the translation start site) of *DoWRKY* genes were obtained from the Herbal Medicine Omics Database or the whole genome sequence of *D*. *officinale* described above. The *cis*-elements were downloaded from the database of Plant *Cis*-acting Regulatory DNA Elements (PLACE, https://dbarchive.biosciencedbc.jp/en/place/download.html)^37^ and used as queries to scan *cis*-elements to test their presence on both strands of 1-k upstream regulatory regions. The positions of both abiotic and biotic stress-responsive elements were marked and shown in a diagram by drawing a gene physical map based on Perl and Scalable Vector Graphics (SVG) script.

### Identification and annotation of potential WRKY target genes

The 1-k promoter DNA sequence upstream of the ATG start codon of each assembled gene from the Herbal Medicine Omics Database was extracted from the genome sequence of *D*. *officinale* downloaded from the Herbal Medicine Omics Database and used to scan for the presence of the WRKY TF binding site element with the sequence (C/T)TGAC(C/T), which represents the consensus DNA sequence of all WRKY TF binding sites that were experimentally verified in plants^38^. To improve the recognition rate between TFs and dehydration-responsive elements, three or more dehydration-responsive elements were proposed to exist in the upstream region, as identified by a yeast one-hybrid method^39^. Thus, the WRKY target genes possess at least three potential WRKY binding sites that were used for further functional annotations using NCBI, Gene Ontology (GO) and Kyoto Encyclopedia of Genes and Genomes (KEGG) databases. For a sequence similarity search, gene annotation was performed by BLASTX at NCBI Non-redundant (Nr, ftp://ftp.ncbi.nih.gov/blast/db/FASTA/nr.gz) with a typical cutoff E value of < 10^–5^. The GO (http://www.geneontology.org/) database was used to perform functional classification to help understand the distribution of gene functions at a macro level by using WEGO software^40^. KEGG (http://www.genome.jp/kegg/), a major public pathway-related database, was consulted to analyze metabolic processes of WRKY target genes.

### Real-time quantitative PCR (qPCR) analysis

Total RNAs were extracted from samples using Column Plant RNAout2.0 (Tiandz, Inc., Beijing, China) and then reverse transcribed into cDNA by the GoScript™ Reverse Transcription System (Promega, Madison, Wisconsin, USA) according to the manufacturer’s protocol. Three independent PCR reactions were carried out for the 63 putative genes using the SoAdvanced™ Universal SYBR^®^ Green Supermix detection system (Bio-Rad, Hercules, CA, USA) according to the manufacturer’s protocol in an ABI 7500 Real-time system (ABI, CA, USA). Amplification conditions were 95 °C for 30 s and 40 cycles of 95 °C for 15 s and 60 °C for 30 s, with a melting curve over a temperature range of 65–95 °C in 0.5 °C increments to check the amplification specificity. *D*. *officinale actin* (NCBI accession number: JX294908), was used as an internal control to normalize the expression of *DoWRKY* genes based on the advice of He *et al*.^41^. Relative gene expression was calculated with the 2^−ΔΔCT^ method^42^. Gene-specific DNA primers for qPCR are listed in Supplementary Table 1.

### Cluster analysis of expression data

The expression profiles via a heat-map of roots and stems were calculated from the log1.5 (2^−ΔΔCT^) value, and shown by a green-red gradient in R version 3.4.0. The data were statistically analyzed using SigmaPlot12.3 software (Systat Software Inc., San Jose, CA, USA) with one-way analysis of variance (ANOVA) followed by Dunnett’s test. The up-regulated genes were defined as a fold change greater than 1.5 with a P-value of 0.05, and a fold change of ≤ 0.66 was used to define down-regulated genes when the P-value was < 0.05. For expression profiles in leaves under cold stress, the raw sequencing reads of leaves under normal conditions (SRR3210630, SRR3210635 and SRR3210636) and treated at 4 °C for 20 h (SRR3210613, SRR3210621 and SRR3210626) were downloaded from the NCBI Sequence Read Archive (SRA) provided by Wu *et al*.^43^. All usable reads were mapped with *DoWRKY* gene nucleotide sequences using TopHat version 2.0.8^44^, and gene expression level was then calculated by the FPKM (fragments per kilobase of exon per million fragments mapped) method using cufflinks version 2.1.1^45^. The genes with FPKM > 10 in control or cold-treated leaves were regarded as valid genes for which fold change (mean of FPKM_treat_/mean of FPKM_control_) was calculated. Genes with a ≥ 1.5-fold change and deviation probability ≥ 0.8 were defined as up-regulated genes, and those with a ≤ 0.66-fold change and deviation probability ≥ 0.8 were regarded as down-regulated genes.

## Results

### Identification of DoWRKY transcription factors in *D*. *officinale*

A total of 83 putative *WRKY* genes were obtained by the HMMER3.0 platform and 81 of these genes were further analyzed to confirm the presence of the WRKY domain by NCBI BLAST. The 81 *WRKY* genes were termed *DoWRKY1* to *DoWRKY81*. The DoWRKY proteins without a WRKY domain and redundant genes were excluded. After this exclusion, 63 Nr *WRKY* genes were obtained and 3′ end RACE was performed (Supplementary method 1). The 63 DoWRKY amino acid sequences are listed in Supplementary text 2. All 63 WRKY proteins contained a WRKY domain and their lengths ranged from 110 (DoWRKY60) to 731 (DoWRKY37) amino acids, with an average of 329 amino acids. Among the 63 identified DoWRKY proteins, 10 contained two WRKY domains while the remaining members contained only one WRKY domain (Table 1). The highly conserved heptapeptide domain WRKYGQK was present in 56 DoWRKY proteins, whereas several variant heptapeptide domains were present in the remaining seven proteins, such as WRKYGKK in four proteins (DoWRKY24, DoWRKY30, DoWRKY68 and DoWRKY79), WRKYGEK in DoWRKY28 protein, WRKYGRD in DoWRKY6 protein, and WRKYATN in DoWRKY76 protein (Table 1). Among the 63 WRKY proteins, 52 of the DoWRKY proteins had a zinc-finger motif of the C-C-H-H type, while the remaining proteins had a variant zinc-finger motif of the C-C-H-C type (DoWRKY3, DoWRKY5, DoWRKY28, DoWRKY49, DoWRKY55, DoWRKY65, DoWRKY66, DoWRKY70, DoWRKY75 and DoWRKY78) and C-C-H-Y type (DoWRKY57) (Table 1).

**Table 1:** Identified *DoWRKY* genes from *D*. *officinale* and their related information.

Gene name	Annotation ID	ORF (aa)	Conserved motif	Zinc-finger type	Conserved motif number	Group
DoWRKY1	Dendrobium_GLEAN_10130608	529	WRKYGQK/WRKYGQK	C-X4-C-X22-HXH(N)/C-X5-C-X23-HXH (C)	2	I
DoWRKY7	Dendrobium_GLEAN_10115484	638	WRKYGQK/WRKYGQK	C-X4-C-X22-HXH (N)/C-X4-C-X23-HXH (C)	2	I
DoWRKY9	Dendrobium_GLEAN_10112830	434	WRKYGQK/WRKYGQK	C-X4-C-X22-HXH (N)/C-X4-C-X23-HXH (C)	2	I
DoWRKY12	Dendrobium_GLEAN_10109483	542	WRKYGQK/WRKYGQK	C-X4-C-X22-HXH (N)/C-X4-C-X23-HXH (C)	2	I
DoWRKY15	Dendrobium_GLEAN_10100432	549	WRKYGQK/WRKYGQK	C-X4-C-X22-HXH (N)/C-X4-C-X23-HXH (C)	2	I
DoWRKY20	Dendrobium_GLEAN_10094986	717	WRKYGQK/WRKYGQK	C-X4-C-X22-HXH (N)/C-X4-C-X23-HXH (C)	2	I
DoWRKY25	Dendrobium_GLEAN_10089661	611	WRKYGQK/WRKYGQK	C-X4-C-X22-HXH (N)/C-X4-C-X23-HXH (C)	2	I
DoWRKY32	Dendrobium_GLEAN_10077269	578	WRKYGQK/WRKYGQK	C-X4-C-X22-HXH (N)/C-X4-C-X23-HXH (C)	2	I
DoWRKY37	Dendrobium_GLEAN_10074853	731	WRKYGQK/WRKYGQK	C-X4-C-X22-HXH (N)/C-X4-C-X23-HXH (C)	2	I
DoWRKY39	Dendrobium_GLEAN_10074607	557	WRKYGQK/WRKYGQK	C-X4-C-X22-HXH (N)/C-X4-C-X23-HXH (C)	2	I
DoWRKY62	Dendrobium_GLEAN_10044500	302	WRKYGQK	C-X4-C-X23-HXH	1	I
DoWRKY63	Dendrobium_GLEAN_10044501	353	WRKYGQK	C-X4-C-X22-HXH	1	I
DoWRKY80	Dendrobium_GLEAN_10003356	330	WRKYGQK	C-X4-C-X22-HXH	1	I
DoWRKY81	Dendrobium_GLEAN_10000561	135	WRKYGQK	C-X4-C-X22-HXH	1	1
DoWRKY2	Dendrobium_GLEAN_10129229	277	WRKYGQK	C-X5-C-X22-HXH	1	IIa
DoWRKY43	Dendrobium_GLEAN_10069437	309	WRKYGQK	C-X5-C-X23-HXH	1	IIa
DoWRKY73	Dendrobium_GLEAN_10020166	302	WRKYGQK	C-X5-C-X23-HXH	1	IIa
DoWRKY77	Dendrobium_GLEAN_10013350	225	WRKYGQK	C-X5-C-X23-HXH	1	IIa
DoWRKY42	Dendrobium_GLEAN_10070674	451	WRKYGQK	C-X5-C-X23-HXH	1	IIb
DoWRKY53	Dendrobium_GLEAN_10059328	570	WRKYGQK	C-X5-C-X23-HXH	1	IIb
DoWRKY69	Dendrobium_GLEAN_10025602	535	WRKYGQK	C-X5-C-X23-HXH	1	IIb
DoWRKY4	Dendrobium_GLEAN_10121280	262	WRKYGQK	C-X4-C-X23-HXH	1	IIc
DoWRKY10	Dendrobium_GLEAN_10112584	303	WRKYGQK	C-X4-C-X23-HXH	1	IIc
DoWRKY14	Dendrobium_GLEAN_10102564	316	WRKYGQK	C-X4-C-X23-HXH	1	IIc
DoWRKY26	Dendrobium_GLEAN_10089597	147	WRKYGQK	C-X4-C-X23-HXH	1	IIc
DoWRKY27	Dendrobium_GLEAN_10085956	256	WRKYGQK	C-X5-C-X23-HXH	1	IIc
DoWRKY40	Dendrobium_GLEAN_10073350	162	WRKYGQK	C-X4-C-X23-HXH	1	IIc
DoWRKY44	Dendrobium_GLEAN_10069083	617	WRKYGQK	C-X4-C-X23-HXH	1	IIc
DoWRKY48	Dendrobium_GLEAN_10063016	187	WRKYGQK	C-X4-C-X23-HXH	1	IIc
DoWRKY50	Dendrobium_GLEAN_10060580	158	WRKYGQK	C-X4-C-X23-HXH	1	IIc
DoWRKY18	Dendrobium_GLEAN_10095806	329	WRKYGQK	C-X5-C-X23-HXH	1	IId
DoWRKY45	Dendrobium_GLEAN_10064360	159	WRKYGQK	C-X5-C-X23-HXH	1	IId
DoWRKY52	Dendrobium_GLEAN_10059569	280	WRKYGQK	C-X5-C-X23-HXH	1	IId
DoWRKY54	Dendrobium_GLEAN_10058347	149	WRKYGQK	C-X5-C-X23-HXH	1	IId
DoWRKY64	Dendrobium_GLEAN_10043009	199	WRKYGQK	C-X5-C-X23-HXH	1	IId
DoWRKY74	Dendrobium_GLEAN_10016910	331	WRKYGQK	C-X5-C-X23-HXH	1	IId
DoWRKY31	Dendrobium_GLEAN_10079755	397	WRKYGQK	C-X5-C-X23-HXH	1	IIe
DoWRKY33	Dendrobium_GLEAN_10076351	444	WRKYGQK	C-X5-C-X23-HXH	1	IIe
DoWRKY35	Dendrobium_GLEAN_10075224	314	WRKYGQK	C-X5-C-X23-HXH	1	IIe
DoWRKY47	Dendrobium_GLEAN_10063175	396	WRKYGQK	C-X5-C-X23-HXH	1	IIe
DoWRKY51	Dendrobium_GLEAN_10059893	224	WRKYGQK	C-X5-C-X23-HXH	1	IIe
DoWRKY67	Dendrobium_GLEAN_10026080	350	WRKYGQK	C-X5-C-X23-HXH	1	IIe
DoWRKY3	Dendrobium_GLEAN_10121855	293	WRKYGQK	C-X3-C-X5-HXC	1	III
DoWRKY5	Dendrobium_GLEAN_10120404	329	WRKYGQK	C-X7-C-X23-HXC	1	III
DoWRKY28	Dendrobium_GLEAN_10083557	182	WRKYGEK	C-X7-C-X26-HXC	1	III
DoWRKY49	Dendrobium_GLEAN_10060697	274	WRKYGQK	C-X7-C-X23-HXC	1	III
DoWRKY55	Dendrobium_GLEAN_10054889	348	WRKYGQK	C-X7-C-X23-HXC	1	III
DoWRKY65	Dendrobium_GLEAN_10041878	294	WRKYGQK	C-X7-C-X23-HXC	1	III
DoWRKY66	Dendrobium_GLEAN_10037978	253	WRKYGQK	C-X7-C-X27-HXC	1	III
DoWRKY70	Dendrobium_GLEAN_10024898	365	WRKYGQK	C-X7-C-X23-HXC	1	III
DoWRKY75	Dendrobium_GLEAN_10014237	264	WRKYGQK	C-X7-C-X26-HXC	1	III
DoWRKY78	Dendrobium_GLEAN_10010985	295	WRKYGQK	C-X7-C-X23-HXC	1	III
DoWRKY6	Dendrobium_GLEAN_10117096	194	WRKYGRD	C-X4-C-X23-HXH	1	NG
DoWRKY23	Dendrobium_GLEAN_10091977	320	WRKYGQK	C-X4-C-X23-HXH	1	NG
DoWRKY24	Dendrobium_GLEAN_10091032	136	WRKYGKK	C-X4-C-X23-HXH	1	NG
DoWRKY30	Dendrobium_GLEAN_10082894	198	WRKYGKK	C-X4-C-X23-HXH	1	NG
DoWRKY57	Dendrobium_GLEAN_10051729	118	WKKYGQK	C-X4-C-X23-HXY	1	NG
DoWRKY59	Dendrobium_GLEAN_10049096	141	WNKYGQK	C-X4-C-X23-HXH	1	NG
DoWRKY60	Dendrobium_GLEAN_10049097	110	WTKYGQK	C-X4-C-X23-HXH	1	NG
DoWRKY68	Dendrobium_GLEAN_10025631	195	WRKYGKK	C-X4-C-X23-HXH	1	NG
DoWRKY72	Dendrobium_GLEAN_10023473	350	WRKYGQK	C-X4-C-X23-HXH	1	NG
DoWRKY76	Dendrobium_GLEAN_10013560	196	WRKYATN	C-X4-C-X23-HXH	1	NG
DoWRKY79	Dendrobium_GLEAN_10007018	196	WRKYGKK	C-X4-C-X23-HXH	1	NG

### Classification of DoWRKY proteins

**Figure 1:**
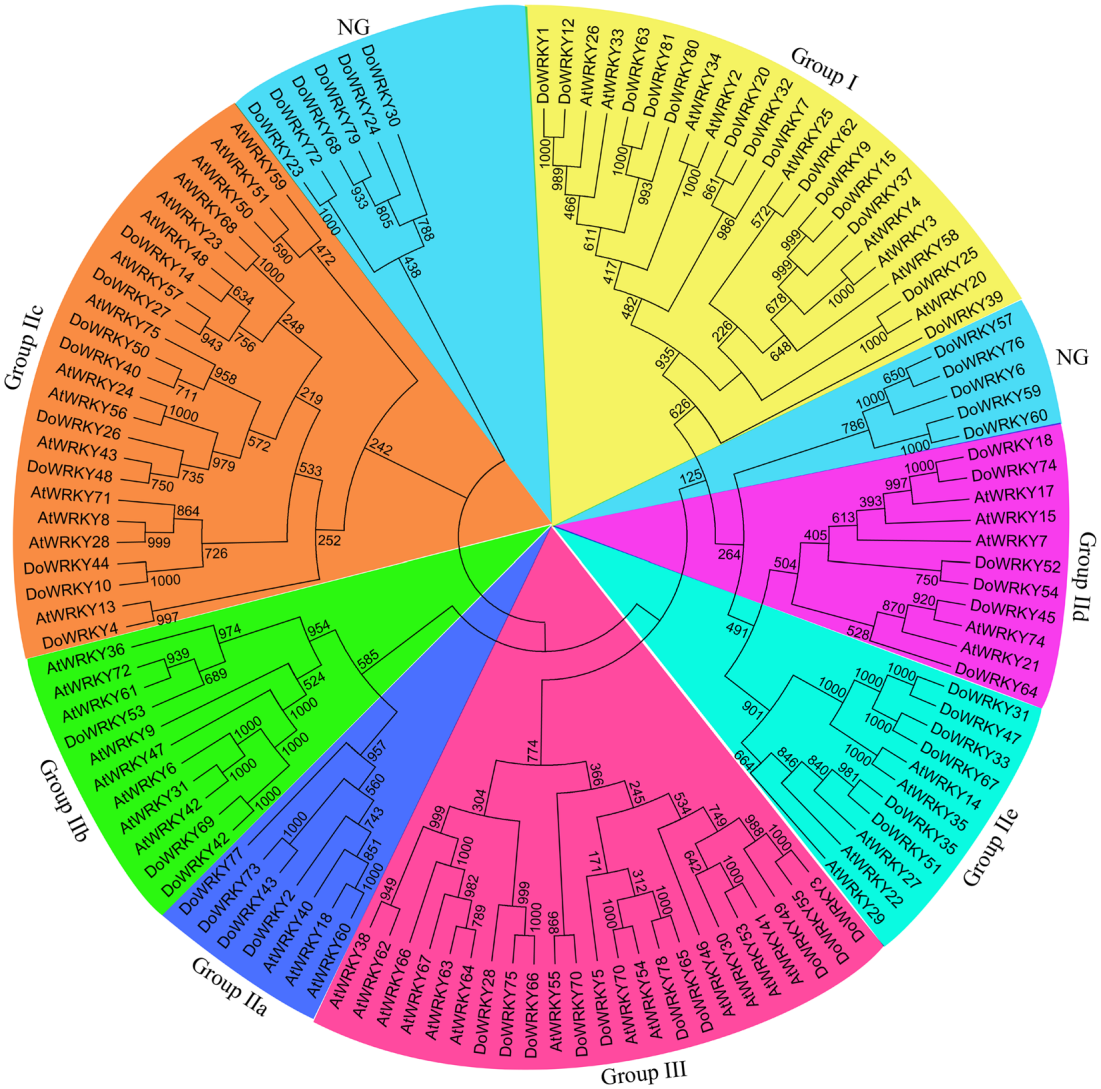
Unrooted phylogenetic tree of *D. officinale* and *Arabidopsis thaliana* WRKY proteins. The 63 DoWRKY proteins and 58 AtWRKY proteins were aligned by ClustalX 2.0 to generate a phylogenetic tree using the Neighbor–Joining method with 1000 bootstrap replicates.

Based on the AtWRKY classification in *A. thaliana* ^46^, AtWRKY amino acid sequences from groups I, II or III were selected and downloaded from PlnTFDB (3.0, http://plntfdb.bio.uni-potsdam.de/v3.0/) to analyze the phylogenetic relationship between the selected AtWRKY proteins and the 63 DoWRKY proteins. The result show that the 63 DoWRKY proteins could be classified into three main groups corresponding to groups I, II and III and into two groups, which were named as the non-group (NG, Fig. 1). Among the 14 DoWRKY proteins in group I, 10 of which contained two conserved WRKY domains (WRKYGQK) and two zinc-finger motifs [C-X4-C-X22-HXH(N)/C-X5-C-X23-HXH(C)], the other four DoWRKY proteins (DoWRKY62, DoWRKY63, DoWRKY80 and DoWRKY81) contained only one WRKY domain (Table 1). Group II could be further divided into five subgroups, IIa, IIb, IIc, IId and IIe and contained 4, 3, 9, 6 and 6 DoWRKY members, respectively (Fig. 1 and Table 1). All the DoWRKY proteins in group II contained a highly conserved WRKY domain and a zinc-finger structure, C-X4/5-C-X22/23-HXH. Ten DoWRKY proteins included in group III had a single WRKY domain and an alter zinc-finger motif C-C-H-C when compared with groups I and II (Table 1).

### Motif composition of DoWRKY proteins

**Figure 2:**
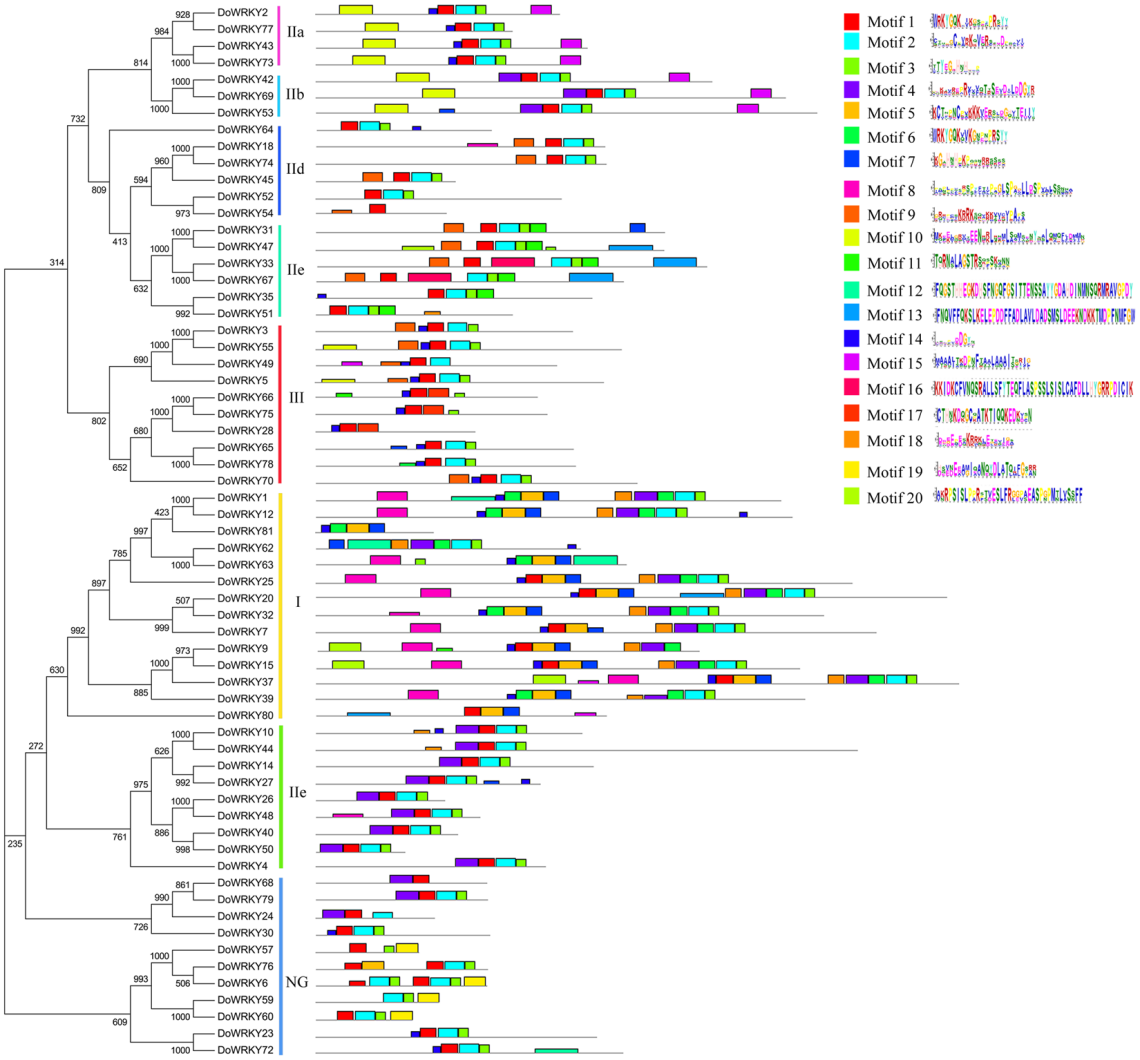
Visualization of the classification of DoWRKY proteins and the distribution of 20 predicted motifs in these proteins. The phylogenetic tree was inferred using the Neighbor–Joining method and 1000 bootstrap replicates with full-length of DoWRKY amino acid sequences by ClustalX 2.0 software. The conserved motifs were investigated by the MEME program.

Generally, members shared similar motifs, indicating a similar function. To better understand the similarity and diversity of motifs of DoWRKY proteins, the conserved motifs of DoWRKY proteins were investigated using MEME online software (http://meme.nbcr.net/meme/cgi-bin/meme.cgi). Among the 20 identified motifs, both motif 1 and motif 6 contained the heptapeptide stretch WRKYGQK, which was regarded as a basic characteristic of the WRKY family. All of the DoWRKY proteins contained either motif 1 or motif 6, or both. Both motifs 2 and 3 had a zinc-finger structure at the N-terminal end and were similar to motifs 1 and 6 for the vast majority of DoWRKY proteins, except for DoWRKY9, −24, −28, −49, −54, 57, −63, −66, −75, −80 and −81 (Fig. 2). The DoWRKY proteins in the same group or subgroup usually had similar motifs, while the motifs in subgroups IIa and IIb were quite similar, with 5 of 6 motifs being the same (Fig. 2). Some motifs were unique in a group of DoWRKY proteins. For example, motifs 6 and 8 were unique within group I (Fig. 2).

### Exon–intron organization analysis of *DoWRKY* genes

**Figure 3:**
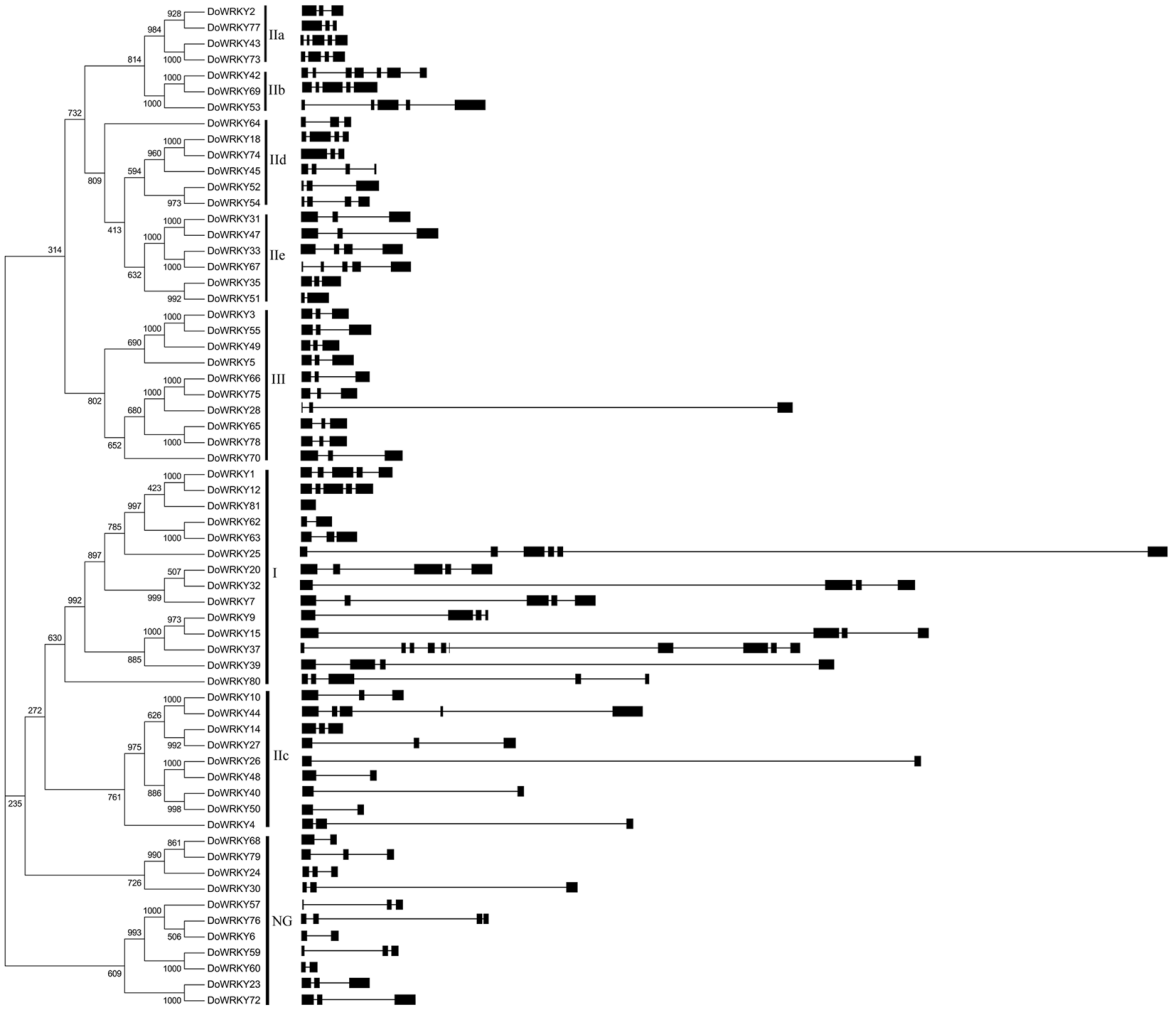
Phylogenetic analysis and structures of *WRKY* genes in *D. officinale*. The phylogenetic tree was constructed by ClustalX 2.0 with the Neighbor–Joining method and 1000 bootstrap replicates based on alignments of complete predicted DoWRKY protein sequences. In the gene structure diagram, black boxes and lines represent exons and introns, respectively.

To obtain insight into the structural features of *DoWRKY* genes, intron/exon distribution was analyzed, as it is perceived as providing a novel source of evolutionary information^47^. Among the 63 *DoWRKY* genes, 31 had three exons and two introns, 10 had five exons and four introns, nine had four exons and three introns, eight had two exons, while the remaining genes had one exon (*DoWRKY81*), six exons (*DoWRKY25* and *DoWRKY60*), seven exons (*DoWRKY42*) and 10 exons (*DoWRKY37*) (Fig. 3). The *DoWRKY* genes that were classified into the same group usually shared a similar intron/exon composition. For example, all the *DoWRKY* genes in group III had three exons while genes in group II had an exon number that ranged from two to five exons, except for one gene that had seven exons (*DoWRKY42*). However, the number of exons in group I varied considerably, ranging from one to 10. This result indicates that exon loss and gain occurred in the groups I and II *DoWRKY* genes during evolution, which may lead to functional diversity of closely related *WRKY* genes.

### Stress-related regulatory elements in the putative promoters of *DoWRKY* genes

**Figure 4:**
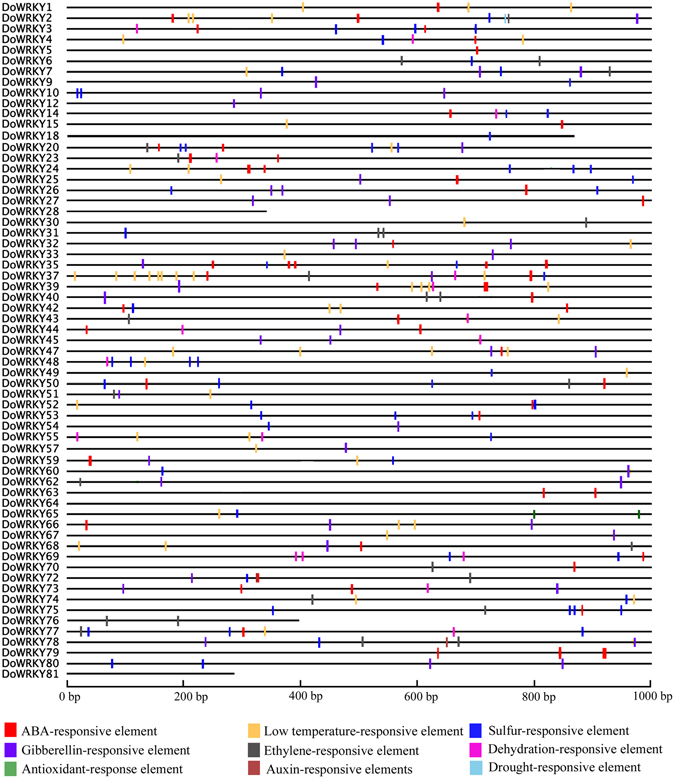
Prediction of *cis*-responsive elements in the 1-k upstream regulatory regions of *DoWRKY* genes. Different *cis*-responsive elements are represented by different colored boxes.

*Cis*-regulatory elements, which are usually restricted to 5′ upstream areas of genes, are the binding sites of TFs, and are responsible for transcriptional regulation^48^. Thus, the 1-k upstream regulatory regions of all the 63 *DoWRKY* genes were used to explore stress-related regulatory elements. As expected, an abundance of abscisic acid (ABA)-responsive elements was present in the promoters of most *DoWRKY* genes (Fig. 4). ABA is known to be a vital mediator of responses in plants to various adverse environmental conditions, including cold, salinity, and drought^49^. Interestingly, low temperature-responsive elements were the second largest group of elements among the promoters of *DoWRKY* genes, which would typically drive genes in response to low temperatures (Fig. 4). DoWRKY37 harbored 9 low temperature-responsive elements in its 1-k upstream regulatory region (Fig. 4). Sulfur-responsive elements, which are known to regulate the sulfur status in plants, were also abundant, suggesting that the *DoWRKY* genes play a role in maintaining the sulfur status of *Dendrobium* plants. Drought-responsive elements and auxin-responsive elements were rarely present in the detected sequences of the 1-k upstream regulatory region, and only DoWRKY 2 and DoWRKY 72, −78 contained one drought-responsive element and one auxin-responsive element, respectively (Fig. 4).

### Expression of *DoWRKY* genes under cold stress in *D*. *officinale*

**Figure 5:**
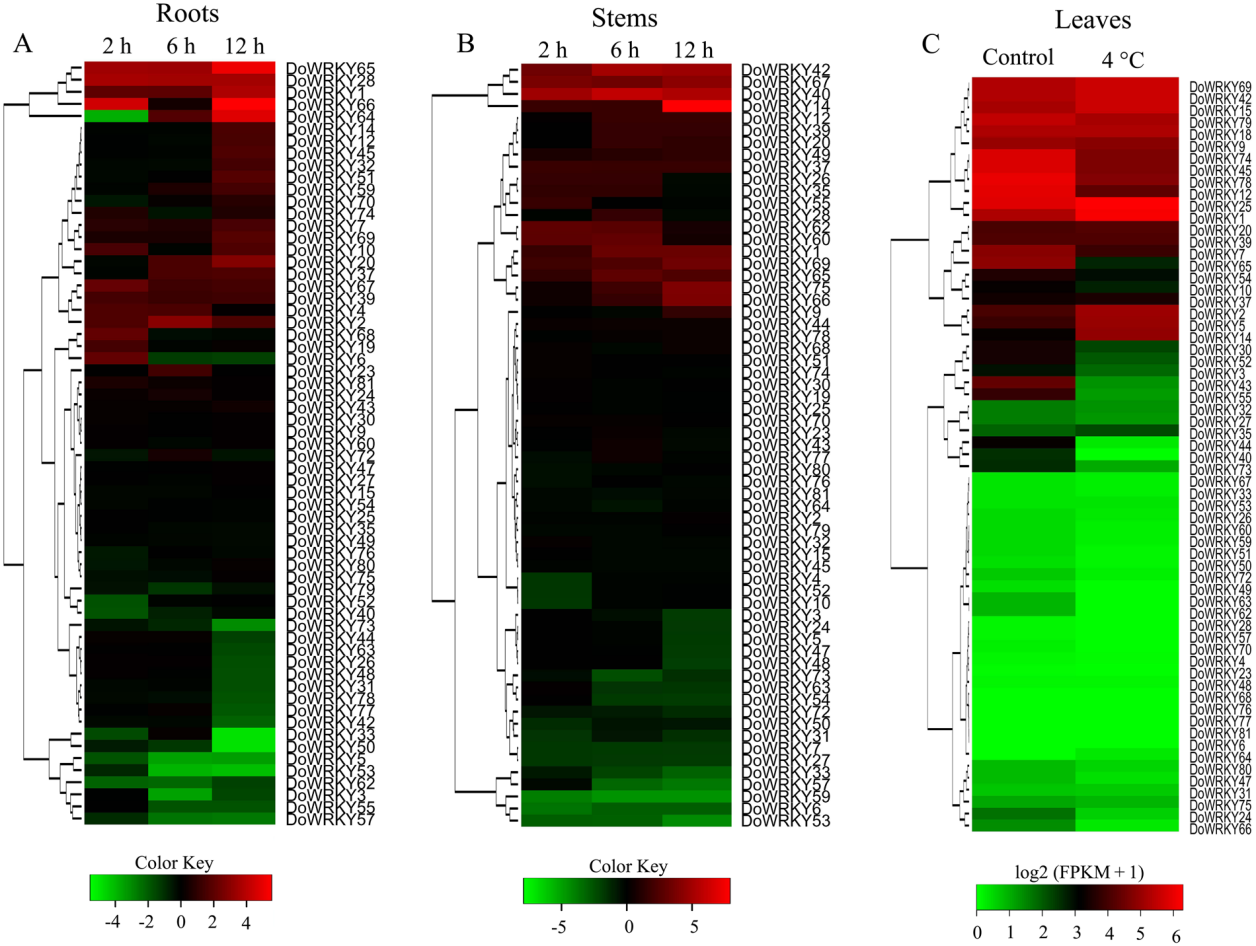
Expression profiles of *DoWRKY* genes with an expression pattern in roots, stems and leaves of *Dendrobium officinale* under cold (4 °C) stress. (**A** and **B**) Clustering of *DoWRKY* genes according to their expression profiles in roots and stems after cold treatments at four time points (0, 2, 6 and 12 h). The expression of the 63 *DoWRKY* genes was assessed based on an analysis of qPCR results. (**C**) Heat map showing expression pattern of *DoWRKY* genes in leaves under cold stress for 20 h. The Y-axis represents the value of the relative expression level [log 2 (mean of FPKM + 1)].

Based on an understanding of the abundance of low temperature-responsive elements in the 1-k upstream regulatory regions of *DoWRKY* genes, a cold stress treatment was applied to *D*. *officinale* seedlings in order to obtain their expression profiles of these genes. The expression profiles of *DoWRKY* genes under cold stress (4 °C) in roots and stems were determined by qPCR while that of leaves was determined by RNA-seq. The data demonstrated that a large number of *DoWRKY* genes were regulated by low temperature in roots and stems. At least two genes (*DoWRKY1* and *DoWARKY14*) were up-regulated in all the organs in which *DoWRKY* genes were detected, namely roots, stems and leaves, while no *DoWRKY* genes were down-regulated in these organs. Six *DoWRKY* genes were up-regulated (*DoWRKY1*, -*2*, -28, -39, -65 and -67), while *DoWRKY5* and *DoWRKY62* were down-regulated at all detected time points in roots (Fig. 5A, Supplementary Table 2). As shown in Fig. 5B and Supplementary Table 3, the expression levels of *DoWRKY1*, -14, -37, -40, -42, -65, -67 and -69 increased at 2 h, 6 h and 12 h in stems. However, just four *DoWRKY* genes (*DoWRKY1*, -2, -5 and -14) were up-regulated under low temperature for 20 h, assessed by RNA-seq analysis (Fig. 5C, Supplementary Table 4).

### Identification and annotation of potential WRKY target genes

**Figure 6:**
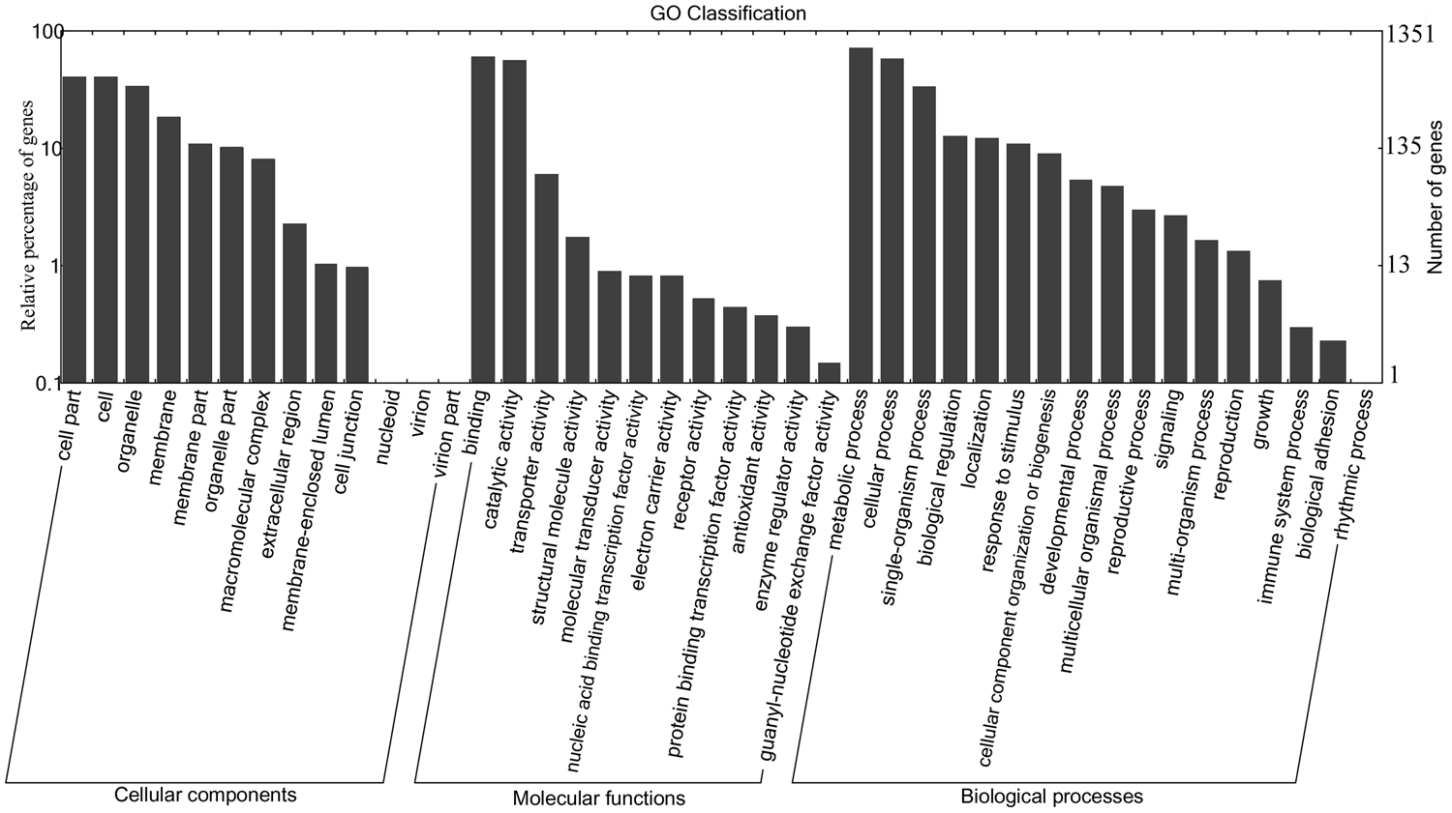
Gene Ontology (GO) analysis of WRKY target genes in *D. officinale*. Categories pertaining to cellular components, molecular functions and biological processes were defined by GO classification.

**Figure 7:**
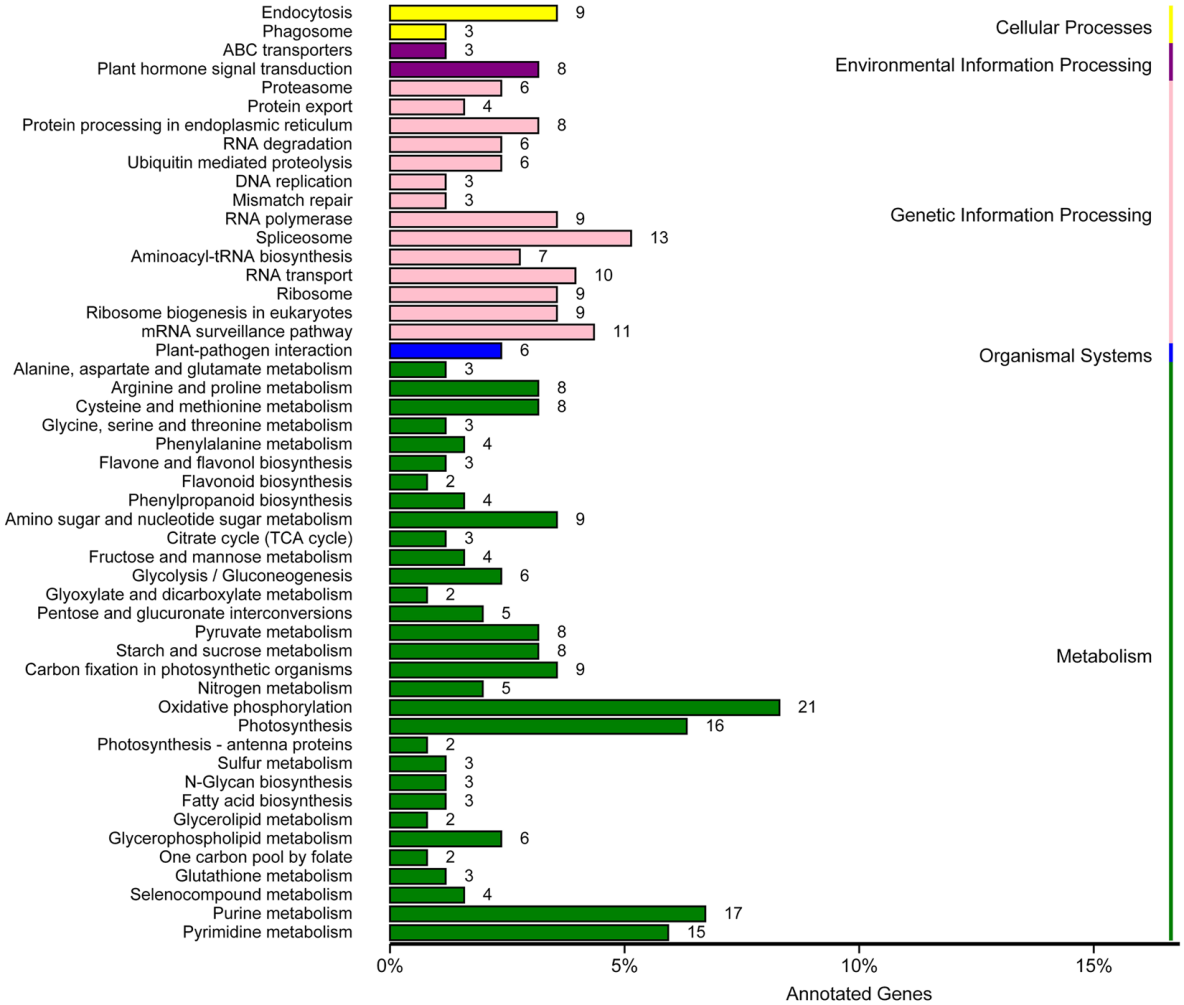
Kyoto Encyclopedia of Genes and Genomes (KEGG) enrichment analysis of WRKY target genes in *D. officinale*. KEGG pathway consists of graphical diagrams contained four main categories: ‘metabolism’ (green), ‘genetic information processing’ (pink), ‘environmental information processing’ (purple), ‘cellular processes’ (yellow) and ‘organismal systems’ (blue).

From a total of 34,417 putative gene promoters from *D*. *officinale* were obtained, 10,757 genes contained at least one W-box element in their putative promoters, while 7127 and 3515 genes contained at least two and at least three W-box elements, respectively in their putative promoters. The 3515 genes with at least three W-box elements in their putative promoters were used for further annotation. Among the 3515 genes, 2504 were related to other known genes or proteins in the Nr database, 1305 were annotated in GO based on sequence homologies, and just 353 mapped to reference canonical pathways in the KEGG database. For the GO classification, the WRKY target genes were categorized into 42 functional groups under three main categories: biological processes, cellular components and molecular functions (Fig. 6). For the analysis of biological pathways, a total of 253 genes were assigned to 88 KEGG pathways, including four main categories: ‘metabolism’, ‘environmental information processing’, ‘genetic information processing’ and ‘cellular processes’ (Fig. 7). More genes were classified under ‘metabolism’ than in the three other main categories.

### Stress metabolic pathways of potential WRKY target genes

**Figure 8:**
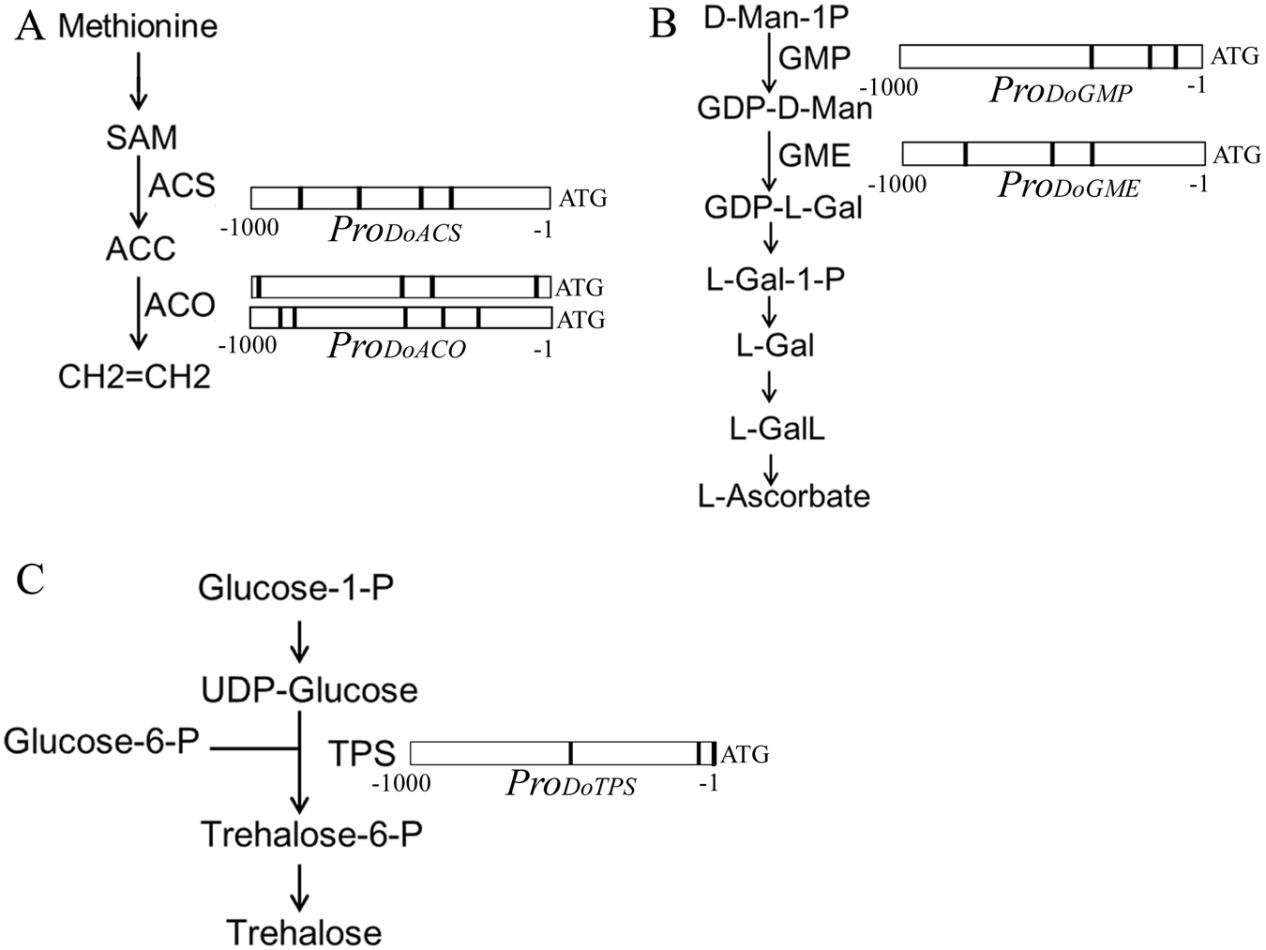
Analysis of the WRKY target genes in the biosynthetic pathway of ethylene, L-Ascorbate and trehalose. (**A**) One gene encoding ACC synthase (ACS) and two genes encoding ACC oxidases (ACO) contained multiple W-box elements in their 1-k upstream regulatory regions. SAM, S-adenoysl-methionine; ACC 1-aminocyclopropane-1-carboxylic acid. (**B**) Visualization of WRKY target genes in the L-Ascorbate pathway. GMP, GDP-Man pyrophosphorylase; GME, GDP-Man-3,5-epimerase. (**C**) One gene encoding trehalose-6-phosphate synthase (TPS) has three W-box elements in its 1-k upstream regulatory region.

The metabolic pathways related to stress responses in plants are shown in Fig. 8. One 1-aminocyclopropane-1-carboxylic acid synthase (*ACS*) and two 1-aminocyclopropane-1-carboxylic acid oxidase (*ACO*) genes had 3–4 W-box elements in their putative promoters. Both *ACS* and *ACO* are involved in the ethylene biosynthetic pathway (Fig. 8A). GDP-D-mannose pyrophosphorylase (GMP) and GDP-mannose 3,5-epimerase (GME), which are both involved in L-Ascorbate biosynthesis, had three W-box elements in their putative promoters (Fig. 8B). The 1-k upstream regulatory region of the trehalose-6-phosphate synthase (*TPS*) gene contained three W-box elements (Fig. 8C). These results indicate that *DoWKRY* genes might play a role in stress responses by regulating stress-related gene expression in *D*. *officinale*.

### Polysaccharide metabolism-related genes may be WRKY target genes

Among potential WRKY target genes with at least three W-box elements, a number of genes related to polysaccharide metabolism were found. For example, glycosyltransferases such as glucosyltransferase, xylosyltransferase, galactosyltransferase, cellulose synthase and mannan synthase, which are involved in polysaccharide biosynthesis, contained 3–7 W-box elements in their 1-k upstream regulatory regions (Table 2). Golgi-localized nucleotide sugar transporters are essential for polysaccharide biosynthesis by providing sugars to the Golgi apparatus^50^. *DoWRKY* genes may also regulate the transcription of sugar transporter genes (Dendrobium_GLEAN_10110460, UDP-sugar transporter; Dendrobium_GLEAN_10127692, GDP-mannose transporter) because W-box elements were found in their putative promoter (Table 2). Mannan mannosidases and glucan glucosidases containing 3–12 W-box elements in their 1-k upstream regulatory regions were identified (Table 2), suggesting that *DoWRKY* genes might regulate the hydrolysis of polysaccharides in *D*. *officinale*. The first WRKY TF was found to bind to the 5′ upstream regions of β-amylase and suppress the expression of β-amylase mRNAs^7^. In this study, W-box elements were also found in the 1-k upstream regulatory regions of amylases (Table 2).

**Table 2:** Identified polysaccharide metabolism-related genes from WRKY target genes and their related information.

Function	Protein classes	Annotation ID	W-box number	Position
Polysaccharide biosynthesis	Glucosyltransferase	Dendrobium_GLEAN_10093863	3	−106, −33, −127
		Dendrobium_GLEAN_10093854	3	−260, −312, −168
		Dendrobium_GLEAN_10034225	3	−905, −49, −110
		Dendrobium_GLEAN_10037085	3	−700, −498, −366
		Dendrobium_GLEAN_10004757	3	−767, −95, −946
		Dendrobium_GLEAN_10114838	3	−449, −46, −580
		Dendrobium_GLEAN_10101470	3	−304, −741, −416
	Xylosyltransferase	Dendrobium_GLEAN_10043111	4	−455, −843, −793, −725
		Dendrobium_GLEAN_10090448	3	−132, −57, −85
	Galactosyltransferase	Dendrobium_GLEAN_10089526	3	−123, −730, −961
		Dendrobium_GLEAN_10090448	3	−132, −57, −85
		Dendrobium_GLEAN_10011963	5	−621, −825, −106, −663, −11
		Dendrobium_GLEAN_10125164	3	−628, −597, −585
		Dendrobium_GLEAN_10117276	5	−928, −582, −220, −158, −304
	Cellulose synthase	Dendrobium_GLEAN_10105279	3	−666, −910, −132
		Dendrobium_GLEAN_10115475	3	−79, −307, −234
		Dendrobium_GLEAN_10037286	3	−366, −531, −157
		Dendrobium_GLEAN_10023561	3	−156, −931, −534
		Dendrobium_GLEAN_10061727	3	−280, −596, −242
		Dendrobium_GLEAN_10064843	7	−577, −516, −628, −536, −857, −527, −127
	Mannan synthase	Dendrobium_GLEAN_10127097	3	−848, −286, −991
	Sugar transporter	Dendrobium_GLEAN_10110460	3	−485, −145, −95
		Dendrobium_GLEAN_10127692	3	−425, −100, −420
Polysaccharide hydrolysis	Mannan mannosidase	Dendrobium_GLEAN_10059324	4	−136, −391, −784, −369
		Dendrobium_GLEAN_10032958	12	−920, −620, −602, −267, −667, −584, −679, −809, −359, −405, −592, −508
	Glucan glucosidase	Dendrobium_GLEAN_10014101	9	−763, −915, −501, −475, −604, −814, −788, −730, −567
		Dendrobium_GLEAN_10108908	3	−271, −536, −41
		Dendrobium_GLEAN_10076876	3	−471, −580, −9
		Dendrobium_GLEAN_10014101	9	−763, −915, −501, −475, −604, −814, −788, −730, −567
		Dendrobium_GLEAN_10108908	3	−271, −536, −41
		Dendrobium_GLEAN_10076876	3	−471, −580, −9
	Xyloglucan hydrolase	Dendrobium_GLEAN_10031220	4	−28, −187, −344, −419
		Dendrobium_GLEAN_10116796	3	−171, −467, −857
		Dendrobium_GLEAN_10095975	3	−299, −32, −95
		Dendrobium_GLEAN_10059366	4	−883, −778,−808, −686
	Amylase	Dendrobium_GLEAN_10097224	3	−146, −937, −909
		Dendrobium_GLEAN_10053987	4	−781, −251, −961, −177

## Discussion

### Identification and structural conservation of DoWRKY proteins

The members of *WRKY* genes range from 48 (*Carica papaya*) to 202 (*Zea mays*) in higher plants (http://plntfdb.bio.uni-potsdam.de/v3.0/fam_mem.php?family_id=WRKY). The number of WRKY genes is not apparently correlated with genome size. For example, only 48 *WRKY* genes were identified in *C. papaya*, which has a genome of 372 megabases (Mb), while *A. thaliana* has over 88 members of *WRKY* genes and a compact 135 Mb genome^51, 52^. *D*. *officinale* has *de novo* assembled 1.35 gigabytes (Gb) of genome sequences^32^ and only 63 Nr WRKY genes were found. As described in the results, the *WRKY* genes in *D*. *officinale* can be divided into three main groups based on a phylogenetic analysis, while 11 of these 63 genes belong to none of the three main groups and are instead subdivided into two subgroups. Group IV or NG were also present in other plants, including rice (*Oryza sativa*)^6^ and grapevine (*Vitis vinifera*)^15^. The WRKY proteins contain one or two highly conserved heptapeptide WRKYGQK and a zinc-finger structure^6^. Of the 63 DoWRKY proteins, at least one contained a conserved heptapeptide WRKYGQK or variants of WRKYGQK. The WRKY proteins have mismatched amino acids in the highly conserved WRKYGQK sequence, as has been observed in many plant species such as carrot (*Daucus carota*)^16^ and black cottonwood (*Populus trichocarpa*)^53^.

### Correlation between the number of W-box elements and the reliability of target genes

An electrophoresis mobility shift assay (EMSA) or yeast one-hybrid system analysis demonstrated that the WRKY TF recombinant protein can bind to the W-box sequence but not to a mutated version of the W-box sequence^54–57^. However, the WRKY protein from *Boea hygrometrica* bound efficiently to the *BhGolS1* promoter with at least two W-box elements, but showed a relatively lower affinity with a single W-box element in the *BhGolS1* promoter after yeast one-hybrid system analysis^57^. A CaWRKY protein showed differences in binding affinity between probes that contained one or two W-box elements^21^. AtWRKY18 from *Arabidopsis thaliana* can only bind to one of three W-box elements but is unable to bind to the other two W-box elements in the *AtABI4* promoter^58^. This suggests that there is a selective affinity of different W-box elements by WRKY protein while the number of W-box elements is correlated with the reliability of putative WRKY target genes.

### *DoWRKY* genes play important roles in response to abiotic stresses

The number of low temperature-responsive elements (Fig. 4) that were present in most promoters of *DoWRKY* genes indicated that expression of these genes may be regulated by low temperature. Seventeen *DoWRKY* genes were inducible by low temperature in the roots of *D*. *officinale* (Fig. 5). Numerous studies have shown that a number of genes from the *WRKY* family are inducible by cold stress^15, 59, 60^. The conserved WRKY domain is broadly considered as a crucial element, which usually binds to the W-box elements in the promoter of the target gene to modulate transcription. The promoters of ethylene, L-Ascorbate and trehalose pathway genes contained W-box elements in *D*. *officinale*, suggesting that these genes may be regulated by WRKY TFs and their products may protect plants from adverse environments. Moreover, many stress-related genes were found to have W-box elements, including ethylene-responsive TFs, NAC TFs, dehydration-responsive element-binding proteins, disease resistance proteins, heavy metal transport/detoxification protein and peroxidases (Supplementary Table 5). Genes from the *WRKY* family confer multiple abiotic stress tolerance in transgenic plants^61, 62^.

### The regulation of carbohydrate metabolism by DoWRKY proteins

The first WRKY TF (SPF1) was identified in sweet potato (*Ipomoea batatas*) where it was shown to act as a negative regulator of β-amylase^7^. Similarly, a WRKY protein inhibited the expression of α-amylase genes, suggesting that the *WRKY* gene acts as a negative regulator of α-amylase genes^63, 64^. In this study, two amylase genes contained W-box elements in their 1-k upstream regulatory regions may regulate by DoWRKY TFs (Table 2). Cell walls are mainly composed of cellulose, hemicelluloses and lignin^65^. Six cellulose synthases and 14 glycosyltransferases, containing 3–7 W-box elements in their 1-k upstream regulatory regions, were identified in this study (Table 2). Studies have shown that WRKY proteins act as negative regulators for secondary wall formation. For example, *atwrky13* mutants exhibited a weaker stem with fewer sclerenchyma cells and vascular bundles, and thinner stems^66^. The WRKY13 protein can bind to the *NST2* genes’ promoter, which belongs to the NAC family that regulates secondary wall biosynthesis^66^. The mutants of WRKY TFs from *Medicago truncatula* and *A. thaliana* can cause secondary wall thickening in pith and are negative regulators of secondary wall formation^67^. A recent study showed that *PtrWRKY19*, a homolog of *A. thaliana WRKY12* in *Populus trichocarpa*, encoded a protein located in the nucleus and functioned as a transcriptional repressor of lignin biosynthesis-related genes^68^. Thus, WRKY TFs might function as negative regulators of carbohydrate metabolism.

In conclusion, a total of 63 *WRKY* genes were identified from an orchid plant, *D*. *officinale*. The classification and conserved domain of DoWRKY proteins, as well as stress-responsive elements in the promoters of *DoWRKY* genes were analyzed. Seventeen of the 63 *DoWKRY* genes were inducible by cold stress, indicating that they may play a role in the cold stress response of *D*. *officinale*. The WRKY target genes were investigated. Multiple W-box elements were observed in the promoters of stress-related genes and in genes related to polysaccharide metabolism, suggesting that *DoWRKY* genes may be involved in the regulation of abiotic stress response as well as in polysaccharide metabolism.

## Electronic supplementary material

Supplementary material
