# Supplementary material for: A Genome-Wide Identification of the WRKY Family Genes and a Survey of Potential WRKY Target Genes in Dendrobium officinale
Source: Sci Rep. 2017 Aug 23;7:9200. doi: 10.1038/s41598-017-07872-8 (PMC5569039; doi:10.1038/s41598-017-07872-8)
Supplement: Supplementary file 1 — Supplementary material [file 41598_2017_7872_MOESM1_ESM.pdf]

A Genome-Wide Identification of the WRKY Family Genes and a Survey of Potential WRKY Target Genes in *Dendrobium officinale*

Chunmei He[1] · Jaime A. Teixeira da Silva[2] · Jianwen Tan[1,3] · Jianxia Zhang[1] · Xiaoping Pan[1] · Mingzhi Li[4] · Jianping Luo[5] · Jun Duan[1*]

[1] *Key Laboratory of South China Agricultural Plant Molecular Analysis and Gene Improvement, South China Botanical Garden, Chinese Academy of Sciences, Guangzhou 510650, China;*

[2] *P. O. Box 7, Miki-cho post office, Ikenobe 3011-2, Miki-cho, Kita-gun, Kagawa-ken, 761-0799, Japan;*

[3] *College of Forestry and Landscape Architecture, South China Agricultural University, Guangzhou 510642, China.*

[4] *Genepioneer Biotechnologies Co. Ltd., Nanjing 210014, China;*

[5] *School of Food Science and Engineering, Hefei University of Technology, Hefei 230009, China.*

[*] Corresponding author: Tel: +86-20-37252993; fax: +86-20-37252978;
E-mail address: duanj@scib.ac.cn (Duan J.)

Supplementary method 1

Completing the 3′ of DoWRKY genes

Seedlings of *D. officinale* (about 8 months after germination) were used to extract total RNA with Column Plant RNAout2.0 (Tiandz Inc., Beijing, China) according to the manufacturer's protocol. The first cDNA strands were synthesized by reverse transcription PCR (RT-PCR) using an M-MLV Reverse Transcriptase Kit and used as templates for gene cloning (Promega, Madison, USA). The two specific primers were designed for completing 3′ ends (see in the follows table). Then a SMARTer RACE cDNA Amplification Kit (Clontech Laboratories) was used to generate b 3' RACE-Ready cDNA according to the manufacturer's protocol. The 3′ ends products were purified by using the Gel Extraction Kit (Dongsheng Biotech, Guangzhou, China). The purified fragments were cloned into pMD18-T vector (Takara Bio Inc.) and sequenced at the Beijing Genomics Institute (BGI, Shenzhen, China).

Primers used for completing the 3′ of *DoWRKY* genes designing by Primer 5.0.

Primer names	Primer sequence
DoWRKY4F1	5′-ACCCTAAGATTAATAGGGTTG-3′
DoWRKY4F2	5′-GAAGAAGGTTAAAGCACGAA-3′
DoWRKY24F1	5′-GGTGGGAATCTAAGCTGTGT-3′
DoWRKY24F2	5′-CAGATCGTGATGTTTTGG-3′
DoWRKY26F1	5′-GAAGAAGGAGAGCAGGCCGA-3′
DoWRKY26F2	5′-AGGAAGTATGGGCAGAAGAA-3′
DoWRKY28F1	5′-TCAGCGATGGACAATATTGACC-3′
DoWRKY28F2	5′-CATACAGGGATATAAGTTTC-3′
DoWRKY35F1	5′-GCCGTCGACTTTGTCCTGCTC-3′
DoWRKY35F2	5′-GCGGCCGGTCTCCCAGACGA-3′
DoWRKY48F1	5′-AAGGGAACGTTATTAATGGCG-3′
DoWRKY48F2	5′-GTCGACCGAGATTTGCCTTTC-3′
DoWRKY54F1	5′-GCCGCCGGTCGGTGCAGAA-3′
DoWRKY54F2	5′-GTGGGCCTGGCGAAAGTATGGG-3′
DoWRKY68F1	5′-GAGAATGACGAAGTGAAGA-3′
DoWRKY68F2	5′-ATGATGGGTTCAAGTGGAG-3′
DoWRKY70F1	5′-CCGATGTTGGTGGTGGTGGGAG-3′
DoWRKY70F2	5′-GGCGGGCCTGTCGGGTCAACG-3′

DoWRKY73F1	5′-ATGGAGTCAAGGCTCATGTT-3′
DoWRKY73F2	5′-ATATGCGTGCGAGCTGATCC-3′
DoWRKY75F1	5′-CCGCTCTCTCCGACCTCCAAC-3′
DoWRKY75F2	5′-GAGGGTGCAGTCGTGGACGA-3′

Supplementary txt 1 AtWRKY protein sequences used for alignment are as follows: AtWRKY4 (AT1G13960.1), AtWRKY61 (AT1G18860.1), AtWRKY71 (AT1G29860.1), AtWRKY14 (AT1G30650.1), AtWRKY6 (AT1G62300.1), AtWRKY56 (AT1G64000.1), AtWRKY64 (AT1G66560.1), AtWRKY9 (AT1G68150.1), AtWRKY57 (AT1G69310.1), AtWRKY36 (AT1G69810.1), AtWRKY66 (AT1G80590.1), AtWRKY40 (AT1G80840.1), AtWRKY3 (AT2G03340.1), AtWRKY59 (AT2G21900.1), AtWRKY15 (AT2G23320.1), AtWRKY17 (AT2G24570.1), AtWRKY60 (AT2G25000.1), AtWRKY25 (AT2G30250.1), AtWRKY21 (AT2G30590.1), AtWRKY35 (AT2G34830.1), AtWRKY33 (AT2G38470.1), AtWRKY55 (AT2G40740.1), AtWRKY43 (AT2G46130.1), AtWRKY46 (AT2G46400.1), AtWRKY23 (AT2G47260.1), AtWRKY58 (AT3G01080.1), AtWRKY70 (AT3G56400.1), AtWRKY68 (AT3G62340.1), AtWRKY22 (AT4G01250.1), AtWRKY47 (AT4G01720.1), AtWRKY42 (AT4G04450.1), AtWRKY41 (AT4G11070.1), AtWRKY28 (AT4G18170.1), AtWRKY31 (AT4G22070.1), AtWRKY29 (AT4G23550.1), AtWRKY53 (AT4G23810.1), AtWRKY7 (AT4G24240.1), AtWRKY34 (AT4G26440.1) AtWRKY20 (AT4G26640.1), AtWRKY18 (AT4G31800.1), AtWRKY13 (AT4G39410.1), AtWRKY62 (AT5G01900.1), AtWRKY26 (AT5G07100.1), AtWRKY75 (AT5G13080.1), AtWRKY72 (AT5G15130.1), AtWRKY38 (AT5G22570.1), AtWRKY30 (AT5G24110.1), AtWRKY50 (AT5G26170.1), AtWRKY74 (AT5G28650.1), AtWRKY24 (AT5G41570.1), AtWRKY8 (AT5G46350.1), AtWRKY48 (AT5G49520.1), AtWRKY27 (AT5G52830.1), AtWRKY2 (AT5G56270.1), AtWRKY51 (AT5G64810.1), AtWRKY67 (AT1G66550.1), AtWRKY63 (AT1G66600.1), AtWRKY54 (AT2G40750.1).

Supplementary txt 2

The amino acid sequences of 63 DoWRKY proteins as follows in FASTA format.

>DoWRKY1
MASSSGSVEASSASFSFASPYEASFSDLLAGEGSANDRLTGLTVRGFGDRGGGGGGIPKFN
SIPPPSLPISPPPISPSSYFAIPAGLSPAELLDSPVLLSSNNILFSPTTGTFPSKTFNWRGINSGSN
DNEGREEENRAYYDFSFQNQNKRSSFQQQRDAFKGSQQPWNYQESNIKTEIPAANSSEMQ
TLQLNTQVNSNNNVTTEQRKSDDGYNWRKYGQKQVKGSENPRSYYKCTFPNCPTKKKV
ERNFEGQITEIVYKGTHSHPKPQSTRRNSSSSQQPVHPLAIVAVGSAASEHSHGGRLGASNL
DSVATPDDSSVSFGEEDVDLSSHKSNLAGDEFDEEEPDAKRLRREGENDGASAAGNKTVR
EPRVVVQTTSDIDILDDGYRWRKYGQKVVKGNPNPRSYYKCTTMGCPVRKHVERASHD
LRSVITTYEGKHNHDVPAARGSRPPEDNSHGTTTAIRPSAISAAPRNSLFGQRLDANFQSY
NGPFTLDMLSTQRNYGSNYMNQQQLKTKEEPKDDMFLESLLI
>DoWRKY2
MESHSLSLDLTIGIAKQSILQEPSPPPCLQEELKRVTEENMKLTSMLATMCESYDALRAQLI
DMIGPTAAVASSEGARSSSPPSAKRKSTESAGDAANSVSSANSCKKAKEEGSKPKIWKLYA
RTDPSDSSLVVRDGYQWRKYGQKVTRDNPCPRAYFRCSFAPTCPVKKKVQRSADDQCLL
VATYEGEHNHYQSSAAEVENAVSRKTAGTADGVGASGAPETKDSRGEREEARQPELRERL
VKDMASSLTKDSNFTAVIAAAISGRFLGISTAQEM
>DoWRKY3
MEKSHSEDLKMLIDVLREGNGHVMELEAHLYNYETFSSETCLLLSSKLQSTFATAIAIMKL
MEQRGLPSLPPSSATDSPRSQSSERTFKEQERREMCKKRKSLPRWSEQVKACSGSAMEIPL
DDGHSWRKYGQKDILGAKFPRCYYRCTYRHSHGCVATKQIQRSDNNSSLFDITYRGAHT
CNHGFQKPSPLTNEQIIHQNQSFKVKTEALSSNEQKSESSPYFSFPLTPKSCLKLESNVFSSS
SNPENYEGLDLEIADIVCASNSTTNSEMLKIDFGVEQFVFDSEFSFDFY
>DoWRKY4
MLPSTAEISLREGLVESHEVPMDQTSHIRFSFQSSSLPPLTCHPLGIIQNRLFASSTDVVAEAS
SSSSPHLKETALLSSVPLRASEAASTLLRSTTNPCWSWSEETTRGPRATVDTHKNIGSMSD
KSIHENLSNYTVNPKINRVGNNLSVGAMRMKKVKARRKVREPRFCFKTMSEVDVLDDG
YKWRKYGQKVVKNTQHPRSYYRCTQDNCRVKKRVERLAEDPRMVITTYEGRHAHSPSH
DEDDQSQPSSMRSQLSTSFFW
>DoWRKY5
MMESGTDMMVSELYQIHELARKLNENLELSHYEICRSLAKEIICAIEKTICTAKSSSTGETE
LAFFNGREFHVDRLNKEFSTDNEPKKSKKRKIMATGTSQVRVSSDPGADMPPDDGHSWR
KYGQKDILGAKHPRSYYRCTHRKTQGCAAMKQVQKTDENSSIFDITYLGTHTCFISPQHI
ASKTSYYNPQQSLLDQQNSLVLHLGFETNLKLKTESSDLNKDPTSSFSFPSTPIEGMNSEN
VLFCSSQRTECCYGVNLSSALMSPTSYESNCFTWPSCEMSSEVDYALTEIISASNSVA
DLPVMDVEFMRDYEEMDAEILFDTSIFFE
>DoWRKY6
MASSSTPPIIDKEEEANEKNIAEERNDRMMAIQEDAYNWRKYGRDWEHYIHNIKMFRTYL
RCKNKNCKARKKVDWPPNDPNNKIISYEGDHNHIINEEQNRVVIPEDDYKWIKCGQQNIK
RIQKKRSYFKCVNKNCKERKKVDWPPNEPNNIDISYEGDHNHRALATGHSENEEVGIEAN
ENDLTTLRLGPART
>DoWRKY7
MAGMDDDVSAFDDWLPSSISPKTLFSDDSSQKTNDGSLSEPRKPGVVVNDSNQDCATGIQ

VSEEYSTELNTYNVPKSSTRGALSKRIAGRAGFGNLTLDTSQIRKTSFDSPSTNDRSPLITIP
PGLSPTMLLDSPNLVSNSLIEPFRSTDKMDCMVCDNIGAAINGDNIDHATEDIDPPLPSFKL
PLLPFSCAESTPALSIHPQQPNPQDGFSTLSKEKGSTDTLNSNHNSNHVPILDDPQEGDDGP
KGEFTSAFLVAPTKDGYNWRKYGQKQVKGSEFPRSYYKCTYPNCEVKKKVELSQDGHAT
EIIYKGTHNHLKSSIPLSHSGNDLQIDELDPNQYASVPIEQCEQSSPYAETPDGADASSFLSN
DDEDEQETHGSASPDRDSDGDETESKRRKLDACALEMNAASRAVREARVVVQTTSEIDIL
DDGYRWRKYGQKVVKGNPNPRSYYKCTNPGCKVRKHVERAAYDVKSVITTYEGKHSH
VVPTARTSNQASSSTASIITTAASPSSCLLQKKAEAARMADCLGRFDTPAPLGPFGLPSRDQ
QLGPAVPNFSYARALQGFNPNVAMAGLGNQTFKMSVLPPLPAYMSQNHPGTVGYVVQTI
MAKEEPAVNSRPPVPGNLSVYHSLSRRPMGP
>DoWRKY9
MSENGGAVSSAAEVNRPSISLPPRSTVESLFRGGPAEASPGPMTLASSFFEPESEYRSFSQIL
AGAMVSPSVSTTAFDARGGGGRERASLNLEHSRPMNLMVAPSPLFTVPPGLSPGGLLDSP
AMFSSNLGHLGMSHQHALAQVTAQASDSHSQMQSQAEFPSSLSSASLIQPISAMNMTSSQ
PMLFITSGKNIHNTESAFASHSDQRSQPAAIVADKPADDGYNWRKYGQKQVKGCEYPRSY
YKCTHPKCPVKKKVERSLDGQVTEIIYRGQHNHERPIPNKRSKEGAAEVNGNTYPDNSES
LQQDDHLNFNRSSDGMVGLSKKEREGVSEQLSGSSDDEELEDEEAEDGEPEHKRRNLETR
VIESSSHRTVTEPRIILQTTSEVDLLDDGYRWRKYGQKLVKGNPHPRIHFRKLSNLMLYSL
RKMYSELAYS
>DoWRKY10
MSGEKLGELFQHDSTIFDPYSLFSLRSGGPQGLESGLPQFSGFLDYLQSSTDFGFTSSLSQE
PFGLIGDRHKVCKAEPRATENVISSVFAGGGTRAMTANSSVSSSSTEAAGEEESDKNEKNE
EKEQEEEDEENKDVKGVDDGADEDEKAKTVSKSRKKGEKRQRQPRFAFMTKSEVDHLE
DGYRWRKYGQKAVKNSRYPRSYYRCTTQKCLVKKRVERSHEDPSIVITTYEGQHTHHYPS
NVRGSSHMLANPQSSMPPSFHHDLFLQTLTPPLQQFNLPANYHELLLLQNSIHPSFINSNQP
>DoWRKY12
MSSSAGSFEASSAIFSFSTPHTTSFSDLLCGADDASTADETSKSVRGFSDKGGGGGIPKFKSI
TPPSLPISPPPFSPSSYFAIPAGLSPAELLDSPVLLSSNNRLLPSPTTGTFPLKAFNWSGNSGN
HYEDKKDVNRAFSDFSFQTQQRLNVFKGSQQSWNYQEPNIKTELSAPNVQANNTIKEQK
KSEDGYNWRKYGQKQVKGSENPRSYYKCTFPNCPTKKKVEKNLEGQITEIVYKGTHNHP
KPQSTRRNSSSSSSQSFQPLVSSAAASEASEHSHGVRSGTAMESVATPDNSSVSFGEDEVDR
NIQRTNRASEEFEEEEPDAKRWKKEGENEGASVAGNKTVREPRVVVQTTSDIDILDDGYR
WRKYGQKVVKGNPNPRSYYKCTTVGCPVRKHIERASYDLRAVITTYEGKHNHDIPAARG
SSNHSANRPPALENAQAITTTIRPSPAVAANSRFGQRFDTFQHSHSPFALEMLPTDQGHYDD
GFSGFGNPSNSYMNHHQQQQQQQQQQQQKQTGNLFPVAKEEPRDDMFFESLLC
>DoWRKY14
MTEKIEEKLDFMTGAISPFSLDAIEPSFGGGFFDLAADIDAGDRGSFGFVDLLGIHDLPQVL
LDTESYGASAAPALESSETQNFPATPNSSSISSSSSEPSPDADPTMPASNPPEAEDESAEHDQ
AKKMGSSDSKGRKKKGQKRQREPRFAFLTKSEVDHLEDGYRWRKYGQKAVKNSPFPRS
YYRCTSALCGVKKRVERSSNDPSVVVTTYEGQHTHPSPIIPRGTHHFSAPSPELVDLHRFQ
LQSSYVLSSQPSNFHPVVTAAPPSFPPPAPRPIPVLGFGERTYCDSATGIGLRDHG
LLQDLIPSDVMMKREQ
>DoWRKY15
MAEGGGGGGGLAAPVEEGAKRPSISLPARFTVESLFRGGVSEASPGPMTLVSNFFAEDPES

ECRSFSQLLAGAMASPAAITAPRKFLVEGEDTAAARTSVETTIADERGRGVGGGGGGERG
GASLNLGHSRPMNLLVAQSPYFTVPAGLSPGGLLDSPAMFSNSAHFGMSHQQALAQVTAQ
AAHSHTQIHSQAEYPSSLSSISLPQQVSTTNITTAQPMQSITSASYKFASALIPHCDQKAQPV
ALVVDKPVDDGYNWRKYGQKQVKGSEFPRSYYKCTHPKCPVKKKVERSLDGHVTEIIY
KGQHNHQKPVPNKRSKEGAAELNGNSDPNNADCRPQGDLSNLNKSSEGMTSLCKRARE
SGHGASEQLTGSSDGEELGDAEAGIDEAEYSGESEQKKRNLETRVIESASHRMVTEPRIIV
QTTSEVDVLDDGYRWRKYGQKVVKGNPHPRSYYKCTNAGCNVRKHVERASTDPKAVIT
TYEGKHNHDVPAARNSSHSTHALAGPANRNNSAPHVVESNNQIINGISDLRNNVHRPIAVL
QLKGEHEMT
>DoWRKY18
MAVDLIGYAKMEDKMSIQDAAAAGFRGMENLIFHLNRHQQKSSSHRNIDCREIAEHTVSK
FKKMISILNRTGHARFRRGPAGVFASSVVTTPPQPSVSLHPTPAMTAPVVQLQGLTLDFTKS
SPVSGASKVDLNIAAATSAKYIKNSFSMSAPMSSTNSSFLSSITGDGSVSNGREGGSSSVLL
APAPAISAGGAVRSKPPLSSACKKQCPSHAHSEDIAGRFAGGRCHCSKRRKNRVKKTIRVP
AISSKMADIPSDEYSWRKYGQKPIKGSPYPRGYYKCSSLRGCPARKHVERAPDDPSMLIVT
YDGEHRHSQPSDPIVSDNTHCKI
>DoWRKY20
MAGIDDSVSIFADWAASNPSPRTFISKFFNEDTCSRPFPDLGDNTHFGFAEPENHKSAMVN
NEEVEKPALVHSSDIFLEPVQFNVSKSSSKGALSERMAARAGFSTLTVNTSRNGTESVIYSP
SNKRCSYVLLSPGVSPTTLLESPVFLSNAMAQPSPTTGKFPFARAEDSNMFSTLISEETDKT
NVQLIEDVHADAFTFPCLGSSFPFLSPSENKFALSIEEERTLVNMGSSLQPDLKQPVCFTEA
SETKEIAEDVKADSFLANESSPPDDNQNVETDPKGGEFSSMASSAAEEDGYNWRKYGQK
HVKGSEFPRSYYKCTFVNCPVKKKVERSLEGHVTEIVYRGAHNHPKPPSNRRSSTSSSQSF
SDTQMDNSDYHVINANFDGKPTLVNAQNGNGGSEWRGDGIEAVSSTSIAEFATSNSMQTI
SGSRYDGSEALNLPSAMSNDEEEDDGATHGSFSMGCDAEGEEMDSKRRKLDLCTIDMSA
ASRAMREPRVVVQTTSEVDILDDGYRWRKYGQKVVKGNPNPRSYYKCTNPGCTVRKHV
ERASHDLKSVITTYEGKHNHDVPTAKTSNHQSTGLSNSTATGIHPTHSLHRKAEPIQEILMR
FENHAPVDPFALPSRDPQFGTAAGFAFGFGHQGLTSLGIGSLGPMGPLRMPAFAPVQSYLE
HQRKVDDGGFIIPKGEPKEEPILESDLPSIANAPKGYYQFANRLSLGPYL
>DoWRKY23
MMNELEELNRVERSIGDQTGDEFTMQELLYSSDLPLLPPWKFTEREPLSPSKLISKMLHGS
PMKARMSMSCSNGEFDGLSNYRPAIALPEKGLTAKMENKYTLRIKTYGINGVADDGYKW
RKYGQKSIKNSPNPRSYYRCTNPRCNAKRQVERSTEDPDMLIITYEGLHLHYTYSHFLHS
QAHDLSITKKPKTNSVDVQPRLFDQDQQLQTIVLQEPLIIGRDGQQDNNLVEVEGFGKSA
MGDEIMQEETLYNNNKNNRHCSQGLLEDIVPLMVRKPSSPTTSSNEPSYPSQASSPSYSSS
LCWTPETFHFAESVLSSIV
>DoWRKY24
MCRSCVQKNNHRLKSTRVSFKTKSDRDVLDDGYKWRKYGKKMVKSSSNPSMFLGHLQP
NGILWFACSSRDCPTERQIERCFRVLNSATPKEKTQKAFLFGVRVEMFGNWKHAIGKWCSI
RLEMPLHWQKECISRAI
>DoWRKY25
MSEMSISGAGDHVASSIPPEISTTSAGARYKMMSPGRLPITRSPCLTIPPGFSPGALLESPVLL
TNMKAEPSPTTGTFAMPLILNNGGLAALGSPKDSSKGCVSEDGSSPYFEFRPHIRLSSRPGL
SSLAPLGSLGLNSKQQEFVEKNQCLCDTNGSSSSQVVKTECIAQSSPESTWSATASGSPVE

VFTLKANGFCEDASNELQLTKDLDCRIQASQSDQNGSNPLNAVEKSSEDGYNWRKYGQK
HVKGSEFPRSYYKCTHPNCQMKKQLERSHDGHITEIIYKGRHDHPKPQASRRSAVGGVST
GQEEEKEDGLSCFANFEEKFSNAHCHSSHVNPNCNSELSPVSVSDDEVEVGGGQSGLGGD
DAADDDPESKRRKMEVATIDANSIAKNNREPRVVVQTVSEVDILDDGYRWRKYGQKVVK
GNPNPRSYYKCTNPGCSVRKHVERASHDPKAVITTYEGKHNHDVPAAKTTNNNNRESSPP
LITDANIPRTYITHQYSQPEENNAINLHLGVGVGSNHNNLLNEKQHLSGLERIPSHHHHHH
HHQYISSDCNNVMIQLNPVSKLYGSSNNVVFGSRDNKGEGFSFSTQMNNSSSHYYPRADN
LVMGP
>DoWRKY26
MEPQQNFPPILSQQGPPFPILPEASGLHFCSSSSILGGDEQNKGKGRGKKESRPRFVFQTRS
ESDILDDGFRWRKYGQKKVKNCSFPRSYYRCSQLTCNVKKQVQRLSSDAAIVVTTYEGV
HNHPCEKLMETLSPLLNQMQFLSQFQ
>DoWRKY27
MENKIDSTTGDPTWDFSSDGCGGIAYLYGEGDGGGHGWAPPRIHEEDPDTWCSLPPPDLS
EASGSAAARSGGDAVSSSSSEGPPADPGDKPPADSPNNKTSKKVQKRVRQPRFAFMTKSEI
DHLEDGYRWRKYGQKAVKNSPFPRSYYRCTNSKCTVKKRVERSSDDPSIVITTYEGQHCH
HTVNFPRAAAIGFHDHIRTTSSLLENISPPPQQQQQQLYFPATTSLQSVPELPTVLPNDDGLL
GDIVSPGMRGRR
>DoWRKY28
MNCTEPCKRRRVQRWTILTSVPHFDGYEWRKYGEKNIQGYKFPRSYYKCTHNKDQGCPA
TKTIQQKEDNDCNPPKYEVIYSLHHICKTMGTNTHLDKKYSSAPINNTSSTTTTSSCQANS
SQPLLTLVADQLCPMESMLLSAEPQIEENGGIENSNTKSSPSTDLDFDSEWAKWLLSCTEK
A
>DoWRKY30
MEEGSRIAFRTQTDEENLDDGFKWRKYGKKSVKNSPNPRNYYRCSTEGCLVKKRVERDN
EDPSYVITTYEGIHNHSSPGVVYYATQDSVSGRFHLSGEKWKEKNIALKVVDSKSDQSQL
KNEDGIFLMIRQFRSFLKRKQRRHQQWIKGKFNKNVKASNDVIYYECKKLGRIKSECLNL
KANPNKEKVDEKNSVKKGN
>DoWRKY31
MCDYFWQRMDQNNQGDLSDIFRNSSSHTSNDSWHPLPEPAGAFAPPSNLFGDPFAEIGDP
LLGEFATTNYFNSSETTIVKTNSTGRSDDCGAQKMLLEEEGKSSIFSRMLQISPVIPSKMSA
EIGPSPLVGTMAGDSSGCLQISSTRVQGIKRRKSQVKKVVCIPAPAAASGRPSGTGEVVPSD
LWAWRKYGQKPIKGSPYPRGYYRCSSSKGCSARKQVERSRTDPNMLVITYTSEHNHPWPT
QRNALAGSTRSHPSKSNNSASTSKNSPHNLNQLATNIKEEHKECSSSTINLVKEEEAYEME
KALVDPQDHDFDQVFDESTFMMSDSNQPDDFFADLAELESSDPMNLIFPKGFMGDHDHA
KLDEERERNKALDPFNMFDWEGGSSIEETKRGL
>DoWRKY32
MDFEKKKPPISFEEGGKKMGLDCTSDDSSELIGFNVQKSCLKGVHNEKMAEMARLNMLK
AFVSQSDQENIMFEINSSQNESKNLISSLPEESSSYMLLSPGVNLSSMEYLDNFSVSLQALP
DNNASLQENFELLEPFGRKEKTHDIAANQKASHFLDVNGSPPPPPPDDHQNAEKGVNVEF
SSFPESDPTKDGYNWRKYGQKQLKGCEFPCSYHKCTHPNCPVRKKIERSLQGHITEIIYKG
SHNHPKPPPNHRSSFSSSKQLHGPQSVSADHPGSVANFKGNPSLASSYDGFKGPKWSDGF
GGTSSASFTEFNDPSNVLKSSQLELESMESSSCVISIDKEDNEATHSSLSADYNIDQEEMES
KRRKQELCSIGRNTTSRAMRDRRIVVQIPSDLDIPYDGYRWRKYGQKVVKGNPNPRSYY

KCTNRGCSVRKHVERASDDLNSVICTYEGKHNHDVPSARNINLARYFPSSLAETAAMQLP
GLYTRPELAQDSFMSLENCAPICTYGLLGRGKQLEPTTSFSFGFDQSNLASLCPSGSDLMR
RFKMPFFPPIHQNMDRQVSSGNIIPMREPKNEPM
>DoWRKY33
MCDYFWKRLENSQGDLTDIFQGSGGAALAGSAVAADIPTTITDHTWQPSPDPATALPLQN
NCFLDNFANIKDPMLHELAGTGFFGSSEKTAPSNNLSSMAITVIRQPPPLVGLTDTMKVGG
DVAISLPQTPAVKRRKNQARKVVCIPAPAVASNKPGGEVVPSDLWAWRKYGQKPIKGSPYP
RAEAIELGGTGTEVKEKKIDKCFVNQSRALLSFYTEQFLASPSSLSISLCAFDLLHYGRRPD
ICIKSRNLEGRANFKKKGYYRCSSSKGCSARKQVERSRTDPNMLVITYTSDHNHPWPTQR
NALAGSTRSQSSKNNNNNASSSKSSSINQKLPSESITVREEEEEEEAKLISPISSSFPFVKERK
DMENSTVVEHLHHDFNQVFFQKSLKELEPDDFFADLAVLDADSMSLDEEKNDKKTMDPF
NMFGWDGGNSFGVTKRG
>DoWRKY35
MFAMEKNDDGWDLFAVVRSCRAASQPPPPPLPTAKPAEAPSTSFYNEVEEFCKPFFSMVQ
QPKPKPSTLSCSSSLAGAAARAAPRRPVSQTKYRSKKRSLRRILRWNQQKRVVCEVPADG
LAADMWAWRKYGQKPIKGSPYPRGYYRCSSSKGCLARKQVERSRTDPAMFVITYTGDHN
HSLPTHRNSLAGSTRQKFSSAASVGEEGSRTVCLPSTPLKREESGEEEEEEEEEDDDDEGIL
LVEDMEVVAEDEVLFMGLEKLQDSPVAAAVTTGAVSEFFGGESCCFEDSFFLHSWSEESSS
SGATAAAGGCNT
>DoWRKY37
MQQFDGNGRMLQRVSTKLSVSTYGEDDLISLIQTLYIKQPPSSTMVSGYDSEPEWLEYKIK
ETNMLTVDKYQQIGFFPTEKAFSLRYQTAGMLETVLRAGVLGEDDIGEESPRNLKLPSRFP
SVVCENCLYSLKEDMRIRAFHIMDPKGVVDMLLIFLEQRDDAPSSISSFSLQDVPDRIIPFL
GKWKGHSITKRSGVYGATIAEADTVAFLELDENGQVVQMRMVRFLCSNSMAENIRTEAT
GHTEAKRPSISLPPRPSDESLFRLEPAEASPNPMNLVSSFFSEDPESDCRSFSQLLAGAITLPE
TRTTAEAATGEGRGRGGGLNLGQSQAMNLLAGQSPFFMAQSDLSPGGLLDFPAMFSSNL
MQGQLGLAHQQAAHSQLQMHLQSENPSSLFSASLSRSFPTVKMPKVLHMPATTLDSNAY
CSEVASASHSDQVPFPVPIVVDKPVDDGYNWRKYGQKQVKGSEYPRSYYKCTQPNCPVK
KKVERSVDGQVTEIIYKGKHNHQKPPSNKRGKEANMPNGSAELNGHIGFPTNSESHSQGN
AITGLSKRDRESGNVAYDQLSGSSDGEEVGDHEGMDERDYGESDQKKRNLETKLIESASH
RTVTEARIIVQTTSEVDLLDDGYRWRKYGQKVVKGNPHPRSYYKCTNAGCSVRKHVERA
SKDPKAVITTYEGKHNHDVPAARNSSHSTAAAASNSSKPQNIHGRSDPLNNVHRPIAVFQL
KEEREIM
>DoWRKY39
MSTKEAKTDASRKKPSGEVGGDFIRKETAVAVKLEESQPKSDREVDGSILLPAPANETLAG
RKSSAASPIVAVPSTSAGDCRSFLQLLAGAMASPAASSRPPPLLAVPIDAPRVPVVAVPCFLA
PAALLESHGITGQFSMSHQAVLATVTAQAQMQLQAGYPSPSGSLKNSVPHSMLPPMTPSP
LQQRLPLASTEAASSLETEQTPSYDQKSQSAFIVSKTSSDDGYNWRKYGQKQVKSTDRSR
SYYRCTNADCFAKKKVERCPDGQVTEVIYRGQHSHEQPHKTKLSKERGHPSSGPFGVTEG
LDIPGIVTVESDPSTSKIDQNSSIDNPEQQLYCSSDCEGDGSNKAGEDPDDEPEPEPKRRLSL
ITVTNPPPVFRTVKQPKVVMQTAVAGHVSDGYRWRKYGQKIVRGNPNPRSYYRCTHDGC
PVRKHVEKSSYDAKSMVITYEGKHNHDVPSLRYGLDPLPTALFIDSAPTAPAAETLNEHES
KGDKIPSISNPPEIEKQLTGDKVLEFGDEKGLESAQALLSMSCDPSSGEEEGMKSQLFSEKS
AAVPVQNS

>DoWRKY40
MLKKASITMNKQEDISTKNYLLPNFTNGFPGFRDSINTTMKKVMEGGNEEEEKNGSSVG
KKKGEKKAKRPRFAFQTRSQVDILDDGYRWRKYGQKAVKNNKFPRSYYRCTHEGCNVK
KQVQRLSKDEGIVVTTYEGIHSHPTEKNSDNFEQILSQMKIYTRS
>DoWRKY42
MGPVDLPMSLASPTMEADEDRRVVNEMDFFSLEKSRTETEQEAYKTQNLNLEKEDLTMD
TGLHLLSGSDLSMGDGFLVAAQETDEKKSEITAMKAKMGQMEEENQRLRNILAQMNQN
YSFLQMQFISMMHKNKSTKGNTNLSNEELNKLEEGREGIVPKQFLEGRCSTEGNFNGDYC
ALKSSFNGDQEEGKETVSILSSSSSKSCEQAQEATMRKARVSVRVRSDAPMITDGCHWRK
YGQKMAKGSTCPRAYYRCTMAVGCPVRKQVQRCADDQTILITSYEGKHNHPLPPAAMS
MASTTSAAASMLLSGTIASSDSLMNPNLLAQAVLPCPSGFATISASAPFPTVTLDLTQPPNP
LQFQKPSSQLQFSIPAIGRPTAIPQIFGQSLQSEEQQSIADTVSAATAALTANPNFTAALAAAI
TSIIGGGVGGSGGGAHQTGSQACVNDDTPS
>DoWRKY43
MDPSPQLGCSTVNLDLSVGPTNFLIEAPICSRTLATNPIQIRRNSPSREEAEDMKLKVQKLS
EENKKLNEKLSAMSVNYSNLQNQLLDLMNSSPSEKGLTSSPTRKRKSISQESDENSGFIPH
VESMSSEDSYQRLRVDSKSSISKICVKTDPSDRTTLVVKDGYQWRKYGQKVTRDNPSPRA
YFRCSFAPSCPVKKKVQRSVDDLSILVATYEGEHNHRPPLQGETPSRASHGVSSTPNSAGQ
TVTVDLTDDRWHVNSDKVSTETTSLEFQKVMIEQMAAALSKDPNFQTSLANAISERILELP
SSHN
>DoWRKY44
MSGDNKEFFHYESFFDHNYPFFSQHSGGGSGDPQGFEAPPLPYGGFTEYLQSSADYGMLS
KAFDLSFSTKEEAFGVPALGKESEDAVIAGVSTNENSSVMAGVGVMSVTANSSPSSSSVEV
AGEEDYGACEKDRKKNEEAGEEENGGEKDKNITKQKKKGEKRQREPRFAFMTKSEVDH
LEDGYRWRKYGQKAVKNSNYPRSYYRCTTQKCTVKKRVERSHQDPSIVITTYEGQHTHH
CPANVRGSSHLLANQSSAMAMPSFRAELFGRQLTQLGLANPSASSGYGFLQNLAAPSQQL
QISNYGALLQDIMPSFVKSSTEKKYLVNHSQKKRNRATIRKVPQLTTVRKASGSAPDDKCI
VKNLNSSDALISVNLNIFPDKSDIGFLENLDMVGSLMPISPIKVVVDSENAGGIIVGGTVPS
SVNSPLALSSKNREGGNLILDRSTSDHNSNDGGGQDAIMVCESISLRPDVVFPLGTFDLPV
EVRVLCNDLALNDVAMEVMPQVDEPDGTLHECNVSHGVVAVDPSSNNFYVAQGNFGDK
LMNVGDNELCGVNMGVVNIYSSELNPSIFFPVTLPYLVVLVSSVGLRVNEQLVDVPIALLY
ANALYTHMGVRSGKKC
>DoWRKY45
SLSMDASGSVSGLDSKSFNLIGGPQSSEPSNLHSNPRRRCTGREDGSGKCASSGRCHCSKK
RKLRLKRSIKVPAISNKLADIPPDEYSWRKYGQKPIKGSPYPRGYYKCSSMRGCPARKHVE
RCLEDPSMLIVTYEGEHSHNSYLSYAPSAGRTLEVLT
>DoWRKY47
MCDYFNRRMEKSQGDLADIVRNRGLISPPESANELLGAANSWQFLPEPSAVFPRLDDKVF
GGDSLADAADPLLHELARTSFFDSTEASGVQKKMLWEEGNRPNGSLLARMPQISLGKGK
GSPVIGPPQLLAPGGGGPVEISSPRLIAGIKRRKSQAKKVVCIPAPAAASGRPSGEVVPSDL
WAWRKYGQKPIKGSPYPRGYYRCSSSKGCSARKQVERSRTDPNMLVITYTSEHNHPWPT
QRNALAGSTRSHPSKTNNASSTSRSSLHNHKQFTDMKEEPKECSSSNNSSLHVKEEAHEE
KEKALTDHQDHNDFDSVFDESSFNEQMMSNLNQPDNFFADLAELDPDPMNIIFPKGFMG
DKLEEERDNNNNSKAMDPFIMFDWGEGNIFGEAKRGL

>DoWRKY48
MEQYLEAGDLPPLFPDHNYFSPSPLLPSDFFHQEAALPPSALTPPLLLPNGISASLMEEREK
LREEGEGNVNGVGERREKRKAVSGRGKKVSRPRFAFQTRSANDILDDGYRWRKYGQKA
VKNSIHPRSYYRCTHHTCNVKKQVQRLSKDTSIVVTTYEGVHNHPCEKLMEALSPLLKQI
QFLTTRF
>DoWRKY49
MEINLLINALNQGNEQIRELEAQLDQPNSSEACKSLASQVQAALATAISMAKLLNQPAKAS
ESPLLASESPRSENSEKVFKKRKTKTRLNTQVLLCSPSSAVNDGYSWRKYGQKVILGSKFH
RAYYRCSHRDTLGCPATKQVQRSDDDSSLFQISLHGDHTCGNHHCRRIPELPSELHNTSPG
AERLMIVSGDLQPGFSLTSPSICGPSNDISAPNLRGSGSEMAEMLSGASWTMSESPIENWD
FKDDFDTLWSSTTIESTMVEFGAEFPFDFS
>DoWRKY50
KSTAKIANKIRKPRFAFQTRSKVDVLDDGYRWRKYGQKAVKNNNFPRSYYRCTHKDCSV
KKQVQRLSMDEEIVVTTYEGVHTHPVVKCTDNFENILNQMQIY
>DoWRKY51
MVCQVLADGLSGDLWAWRKYGQKPIKGSPYPRGYYRCSSSKGCLARKQVERSRTDQGM
FVITYTGEHNHPLPTHRNSLAGSTRQKFPSAGGEGGGGNLPEPSTSAGEGSVRLSPNTPLTS
GTEEDLFRPRKKKKQEGSTDEDEEEEEAEVGEEEDELDMLLVEDMELVGEDEFLFMGFEE
LQVSSSPAVDELKVSSSPAVHELLSWDGEPQQTGTPPWPPAMAD
>DoWRKY52
MEAKEEPQLLLLSPSTKKSRRSVQKRVVSVPMVDADGGRSKGGGAGEAAPPSDSWAWR
KYGQKPIKGSPYPRGYYRCSSSKGCPARKQVERSRMDPTTFIVTYSSDHDHSWPAMSKNQ
RHQQNSPAAATGAATLPESICSKPVDVENNPVSSDNSLATDQDEKLTDLMVEESAGLMIPD
AFGWFAQVSSPSSNSPAAASDAEFFCNPIFGFDDCDKLSGTAVDEEEDALFAGLGELPECS
VVFRRGFLERQWRGGEDAKPCGIEAEESTAAAASWCGSTS
>DoWRKY53
MEAVKKRPLPAKEPRNEVDGGGEADQERFIKVSPARTGERSSTNTSSRPSSNSKDSPTISQE
DQIQSTKAEIGEVKEENERLKNLLSQIVKDYQSLQMHFFDIVKQEQPNLVTKPTDELTAIKT
HEAEETELVSLRLGSNSVTTDHKKQLEEKLINTSSKSKENGHIEEGLTLGLDCKFEGYTNK
NNSECKEVASNPSSDNSIDEMKEEEAVEPCPPNKMLKNLRTGDDEVLQQTHVKKARVSVR
VRCQTPTMNDGCQWRKYGQKIAKGNPCPRAYYRCTVAPACPVRKQVQRCADDMSILITT
YEGNHNHPLPMSATAMASTTSAAASMLMTGSSTTHSSGIPDAFPSSASPIITGVNSGLHNL
NFSLSNNTRTTPFYLPNTTISPSPSYSTITLDLTAPPSSTAQTSQFNRFSSSSSSSSSFSISMPSS
WSSRSGYMSCAAHDQSYKPNYPHDSFYQSYMQNPTSMASMNQNFFTDSIAKAITSDPSF
QSALQAAITTYVGASGSQSGQVLHHAQSYAGNEYSTASGLFTTSVPSSSSAQQGNLMAMS
SSKSTASASPVDHISWENMK
>DoWRKY54
MYAKEEPGTSPELSPYLDPEPSPKKSRRSVQKRVVSLPVPEAEGPRLKSGGEAAPPPDSWA
WRKYGQKPIKGSPYPRGGRGGRRALRRTRRAARIFCYFPARISGPSDTWRGGRETVWIGS
SGGGVWKQRMSGGGGGHTIVPDFCFFML
>DoWRKY55
MESCLVTSDMNMLINVLNQGNEHMRELQAQLDQPFSAEACKLLATKAKSALSSAISLAK
LLEPSCQRPTGFVDSPPSASDSPKSDSSDKVFKELERREMCKKRKTLPRWSRQVRACSNS
AMEGPLDDGYSWRKYGQKDILGAKFPRGYYRCTYRHTHGCLATKQVQRSSEDDFLFDV

TYRGVHTCNHCLQRTPTPSPKEENLIQNQASKPQAHPHQQQLLEQLKTGLRVKTEGFNFD
EQLLESSPSFSFPSTPPNCIKPHNNLFSSASMPENQLMGNNSTTTSAESNYFSLSPCGVNNF
AGGLNLQGFDTEIAEIISAAASTTNSPMLGMDFPMEQIEFETDFPFGFC
>DoWRKY57
MESMASSETTIIDEEQVQEQNIVVAFSNGLFVIPEDDYKWKKYGQKYIRSIQKNRCVNKN
CKTRKKVEWPLNEPNNIDIAYEGDHNYRALVGHSENEEAGIAANQYDLATQMFGYART
>DoWRKY59
MNIHILAELRRLREQIGGNQIAHASAETKNNSIYRAISGEVQGIDDEKVPAIVVPKDEYQW
NKYGQKLIKRIGNKHRCKKKDCGAKKKVEWAPSNPSTVRVLYEGNHNHQCARIDSDEVD
EDAMIQANQYDLATQIFGSRN
>DoWRKY60
MSEEQKGGDDDQIPTITIPQDEYEWTKYGQKLIKGIGNKYRSYFRCKKKDCGAKKKVEW
APSNPSDVRVLYEGSHNHQPARITGSDQVDEAAMIQVNQYDLATQIFGSRN
>DoWRKY62
MVERSLDGHITEIIYKGTHSHPKPQRIRKNSSSFEGFQGSTHHEGKDHSFNGQFGSITTENS
SAYYGDAHDINMNSQRMRAVGPDYDDDEADAKKWKMEGEVEGISISSDRTVREPKVMV
QTTSDIDVLDDGYRWRKYGQKVVKGNPNPRSYYKCTTLGCSVRKHIERASTDLRAVITTY
EGKHNHDVPAPRGSGGLTNKTSFDDNNNIRTNNSSSSSSYNMSNFTMAVRPSVASNSFFSP
RTNGMDGIGFFRYENSMNSLFANELQKQNQLQHLQKQGESVLSKAKKEPRDDLFTDSLL
G
>DoWRKY63
MTPSSSSFVTSENSVPILPFSTSSSSQLNTFLRASEAVGEERSTFRSSNGFKSTVTTSSLFPNA
SSSFSPPPFLSLPGGLSPAVFLNSPIPLSSSNAQILASEAIGSTFPTQTITWTGNNTNYLHELKE
DRYHNKSYSNFSFETSTRPASLKSSSFHPSSIGFMASEEEAFEAANQQSWSYQQPPLITSND
QIPNLQASSQSEYNYNYSNISAQSLREQRRLDDGYNWRKYGQKKVKGTENPRSYYKCTY
PNCPTKKKVERSLDGHITEIIYKGTHSHPKSQRIRKNSSSFEGFQGSTHHEGKDHSFNGQFG
SITTENSSAYYGDAHDINMNSQRMRAVGPDYDDDEADA
KKW
>DoWRKY64
MAQKTVVIVRGEAKLGKHGGEAPPDDWSWRKYGQKPIKDSPYPRAYYRCSSSKGCSAK
KQVERCKKDESLVIISYTSSHNHPGPVPSSLNKSINSSRIPFLNEDCRSSEKNRNVEDNYESS
IVKCAQESTEKVQGEGDTISSSLIEPVNCSMEDDDIFDELDELPLPSSFNSLMKMVYCSLAI
NEADLVVEKIVWHSVR
>DoWRKY65
MASSEAEGSLPPAMALVIDELTKGRQSVATLATLLFVDPLELQTAKLLLEETMSTISKAIAIL
ECVNSQQDHTAVQLESPSSAVTSKGPVGKRKIQSTRKAASRKRGNPYSWKRVTSATIEDG
HTWRKYGQKEIFSAKHPRSYFRCTYKYDQGCKATRQVQKSEEDPFLYVITYFGEHTCEAA
KENNPRCREPCVVSFESNKINEIKQEIAFSSFPSHKQENEEEVVSNLTSVDSRSNCLEQPAM
ELHKSSITGLEPGWSDSSSGLNYSINSYDVEFMDLEDILSFELNEFFAW
>DoWRKY66
MAEHPFSQSSTVIDHLSRGYERLTQLLALISSSPANPSTTAPAMVLVDKAMCCIAAALSELQ
TSSPRSVDSPTAAEDHRNGTDSKKRCKKNVQSWTIVTSVPHFDGHQWRKYGQKRIHSSEF
PRSYYKCTHSKDKGCKATKTIQQKEDKISNNPPKYVVSYSVHHTCKAIETHEIPIVMDSLA
ITNEHSTSSSQAASPDLSSPVLPKPVADIEQVSTIMDYTEPDHGEANWFEGSGLLSPFADSDI

DWERLMS
>DoWRKY67
MSYSSNLPLHAWPPPLVGLGDTMKVGGDVAVSLPQTPAVKRRKNQARKVVCIPAPAVASN
KPGGEVVPSDLWAWRKYGQKPIKGSPYPRAEAIELGGTGTEVKEKKIDKCFVNQSRALLS
FYTEQFLASPSSLSISLCAFDLLHYGRRPDICIKSRNLEGRANFKKKGYYRCSSSKGCSARK
QVERSRTDPNMLVITYTSDHNHPWPTQRNALAGSTRSQSSKNNNNNASSSKSSSINQKLPS
ESITVREEEEEEEAKLISPISSSFPFVKERKDMENSTVVEHLHHDFNQVFFQKSLKELEPDD
FFADLAVLDADSMSLDEEKNDKKTMDPFNMFGWDGGNSFGVTKRG
>DoWRKY68
MAALLEFIDQFTNFSSFHFPEFYSDGQPELDVYDYAEAEDAHLFDESCLTFTGETEAVVAA
ATVVSTGKDGGEQRNGQENDEVKRRKVEAEFRIGFRTKSEVEILDDGFKWRKYGKKAVK
NSPNPRNLQSSANSRCFPKICKSSGVFRRQEELRRWSRRGRKVNFFLTMLELNIFNPWWIP
RAKDLLQRRLSIIS
>DoWRKY69
MENKGGGGLTLEPNPFRAIKRRQSMDSAAGFEFPISLACREAAPSSHDDQKPAGEMDFFSS
EKKARVSTPVGIPLDLMIKKEDLTINTGLHLANTGSDQSTLDEGMSLNEEEKEGKTELVAM
QSELGRMTEENQRLRGMLSQVTNNYNALHMHFMALMQQRNQNNKSNNITQSQEAVSD
DQLRKQENVGVAIVPRQFIDLGLAAGDADEPSTHSSTDGGSQDPSSQSQANKEKSANSKE
MMVFEREGNGFVREESPENSSHVWAPNNQPKVGGVGLKPAEQAQEATMRKARVSVRAR
SEAPMIADGCQWRKYGQKMAKGNPCPRAYYRCTMATGCPVRKQVQRCAEDRSILITTYE
GNHNHPLPPPAMAMASTTSAAASMLLSGSMPSNDGLMNSNVLARTILPCSSSMATISASA
PFPTVTLDLTQSPTPLQYQQRPPPPQFHLPPTQPAQPSSLPHIFGQAIYNQSKFSGLQMSSAE
VARPPPPHNLADTVSAATAAITADPNFTAALAAAITSIIGGTHQAGSSSNSGAVNN
>DoWRKY70
MDEILNQVLQACRLSRELESSLPHIANNPNVLLASCEQVSLAFNKVVYDLRGCLGGSSSSF
DPTGQLIIGERIMEDWSHLHDPDSMPTGQMAFSQFGLDIQVTGAAIAAVEFADVGGGGRL
AGGEQEHAVVVVSEAETGHGGRLGGPVGSTGQRPSRRRRDSGDTRTVRIPALRTGNLELP
PDDGYTWRKYGQKAILGSKFPRSYFRCTHRNFYGCKAKRKVQRLDADPNTYEITYCGNH
TCQTSPTPLILPSMTPTTNESMSGNVAIMTGASPPPAFPLSTSIELSSWFLKEHESEGKAPSR
PFLSSHEVGESSRAHAGPSEQAAPPVRTGREGEGSVADLADAMFNSGSSSNSMDTIFTFKT
D
>DoWRKY72
MALKAGSLLVPYAQSISLLIKLIRMEDMLDESVAKWLESLEEEFNMQDVLFHTEQVPLMA
PQRRATEPKSEVRSAELVNLLISKVYSGPTIGDIESALSLSCSDDGGRNYRSALQEKGSSTK
MDKYTLRLKTCDNGVADDGYKWRKYGQKLIKNSPNPRSYYRCTNPRCNAKKQVERSTE
DPETVIVTYEGLHLHFTYSHILLSQKNDQHAASLNALKKQKKQTELQPKESEGQQHMQQI
AQSIQNREQPQGNKHLRDGESYQEFCLEDILLENPSMREKNIHGLLTDVQRPQGMLEDVV
PLMVRKPCSSATPSNEPSYFSQESSPSHYSSLFISPSSSQIDKGILSSIL
>DoWRKY73
MEPATCLGFSTLNLELSVGPADLTEKGLASKTPPDRQQESMFMKEKLSKIMEDNRKLNEM
LSDMSTNYCSLQNKFFDFMNSSPSEKAAGMASPPTGKRKSDSLESYSHENVNNGVKAHV
ESLPIEFDSCKRLRMDSKSSVSKICVRADPSETSLVVKDGYHWRKYGQKVTRDNPSPRAY
FRCSFAPSCPVKKKVQRSANDLSILVATYEGEHNHKAPLQSEGSGRLSHVGSSITPSLSINSS
SPTIMVDLTNDKLHAEAKKACGEMASTEIQKFMIEQMAAAIAKDPTFRATLANVISGRIF

>DoWRKY74
MAVDLIGYAKMDDQITIQEAAAAGIRSMENLIFQLNRHHQKSTSSSATAATIDCREIADHT
VSKFKKMISILNRTGHARFRRGPSSPILPAAVKPTPQVAVLPSAPAMAVPVVPLQSLTLDFTK
PSSVGGGSSDLAIVAASSGKYIKETFSISTPISSANSSFMSSITGDGSVSNGRPGGTTSLLLPP
APAISAGGVVSGKPPLAAYKKRCHSHAHSEDFAGKFAANGGRCHCSKRRKNRVKRTIRVP
AISSKMADIPPDEYSWRKYGQKPIKGSPYPRGYYKCSSLRGCPARKHVERAP
DDPSMLIVTYEGEHRHSQPSDALVFDTHREI
>DoWRKY75
MEETPYNHSSTAIDNLSQGFEGLTQLRALFLASSPAKPTDKTLAIGLLDKALRCITAALSDL
QPISPTVAVEDRKNDTDHKKRKRVQSWTIMTSVPHFDGHQWRKYGQKRIHNSDFPRSYY
KCTHNKDQGCQATKTIQQKEDKVPNSPIYKVSYCMHHTCKPIETQENPFVMDSSAIMNST
SSTTASSSYSSHELSISSFQAPSSDQPTVFPLQLEESTEQVFPMESMLLSSETRGDLAVLGLD
WEWQDELITLGEFGDLIVRI
>DoWRKY76
MKINSDKEEEAKEKNIAEERNNRTMTIQEDDYKWRKYATNYIHKIKKFRSYLRCVNKNCK
ARKKMDWPLNDPNNIDISCEGHHSHRCMESMASSETTIIIEEQGQEQNIALAFNNALVVIP
KDDYKWDKHGQKFIISIQKNRSYFRCANRNCKARKKVDWPRNESDNLYISYEGDHNHKV
VAVGHSENEESGIAAN
>DoWRKY77
MGSNNGSSICLDLSIGTLQFSNSCMKQHNKINKLPLIHGSTKMEEQEIYSSQIEAEMLDEM
NRVVAENRRLSKMLAAISERHRALRAQVLEMINSTSSSSSTKPAPKRRNCDGSLGYPQSVN
ASYKVNCAKEEVRETRPRISRMYIRSDGLVVRDGYHWRKYGQKVIRDNPYPRAYFRCSF
APVCPVKKKVQRCTEDHSVLVVTYEGEHNHVQPLPGDSLNCLR
>DoWRKY78
MGSQDEEILSPPIWKSAAMKELARGLDSVAKLASLLQVGSSKNGRPEAATAGLLAEEIMC
RISKAMAALESGGNIPSSCVTGNKEGVGGKSKMQSTRKAGYRRSRGSPYSWTRITSNTID
DGHTWRKYGQKDIFSAKYPRSYFRCTHKFDQGCKASKQVQRSEDDPSLYVITYFGEHTC
VDAKKATITPSRELCIISFESSNGENIKQETLTSFPSLNQESEEEVLSNLTSADSASDYFVSPK
VDIKKSEEQTAAELGQEHNGLPLALYTSLMNYGMEFMDYEEFLNFDHDLFQN
>DoWRKY79
MAALLEPAEMNSHFSSLQPAKLLHSSCEGSLAATGGGGGSGDVFYFAQIDQSQFFDGSCLT
EVAGELETPTSRMIESSDLYEHEMEYEELKRRKAEGGCRIGFRTKSEVEILDDGFKWRKYG
KKAVKNSPNPRNYYRCSSPGCEVKKRVERDRSDSSYVITIYEGTHNHISPDCKPYTTNPPQ
LAIAPSHSWNQQF
>DoWRKY80
MQDKNKNSSANLRKSPEQLAGSSSASPSIISEIAFKPKAVRLKPVANLSSEIAFKCDGSVQA
ASMALDMESIDISKENSKTIILYRPIAQENHEEHKQSLLDVHFQPQELNQVNCSQSTMSLH
QSLTSSSNANNFLCSSNSSHESKKTKVKSQQITKNGDRNSLDGYNWRKYGQKQVKGSEY
PRGYYRCTHPFCPVKKMVERSKDGEISEIVYRGDHNHPKSQKKEQISSSRLREQTSVLDG
NGGNFMDDRAVTDFLLEENLFENKTENCDVEDFLDTGINRKALYSDDSDTVLHALSLSFG
ADLNGGKAAAFSTRDGYEKKSQKRYLFY
>DoWRKY81
MAAQALNDQKGVEDGYNWRKYGQKQVKGSENPRSYYKCTYPNCPSKKKVEKTLEGQI
TEIIYKGKHSHAKPKPTRRNSSYFGSDQTFLNSQVLDSITNAENSSASYGDDDFDMNSMM

TKSQGDGFDEDEPDWKKM

Supplementary Table 1 Primers used for qRT-PCR.

Primer name	Primer sequence	Primer name	Primer sequence
DoWRKY1F	5′-CCGTTCGAGGATTTGGAGATAG-3′	DoWRKY45R	5′-CGTGGATTCGAGTGCAGATT-3′
DoWRKY1R	5′-GAGGAGGCGATATAGGAAGAGA-3′	DoWRKY47F	5′-CCAATCAAAGGGTCTCCTTACC-3′
DoWRKY2F	5′-TCCATGTCCGAGAGCTTATTTC-3′	DoWRKY47R	5′-TAGGATCTGTCCGGCTTCTT-3′
DoWRKY2R	5′-CACTGATCATCGGCACTTCT-3′	DoWRKY48F	5′-TTCAGACGAGGAGTGCTAATG-3′
DoWRKY3F	5′-TCTCCTTACTTCTCTTTCCCTTTG-3′	DoWRKY48R	5′-GTGATGTGTGCAACGGTAATAG-3′
DoWRKY3R	5′-CCCTCGTAGTTCTCTGGATTTG-3′	DoWRKY49F	5′-CGACGACGATTCTTCTCTCTTC-3′
DoWRKY4F	5′-CAGACATCCCACATACGATTCA-3′	DoWRKY49R	5′-CCCGGCGAAGTATTGTGTAA-3′
DoWRKY4R	5′-GTCGGTAGAGGAAGCAAACA-3′	DoWRKY50F	5′-TGGCTATCGTTGGCGTAAATA-3′
DoWRKY5F	5′-AGCACTGGTGAAACAGAGTTAG-3′	DoWRKY50R	5′-GCAATCCTTATGTGTGCATCTG-3′
DoWRKY5R	5′-TGGATTTCTTCGGCTCGTTATC-3′	DoWRKY51F	5′-GGAGAAGGAAGTGTTCGTCTTT-3′
DoWRKY6F	5′-GAACTGTAAGGCGAGGAAGAAG-3′	DoWRKY51R	5′-GCTACCTTCCTGCTTCTTCTTC-3′
DoWRKY6R	5′-TTGCTAAGGCTCTGTGGTTATG-3′	DoWRKY52F	5′-GGACGTGGAGAATAATCCTGTATC-3′
DoWRKY7F	5′-TTACCATTCCTCCGGGTTTAAG-3′	DoWRKY52R	5′-CATAAGCCCTGCCGATTCTT-3′
DoWRKY7R	5′-ATGCAGTCCATCTTGTCAGTAG-3′	DoWRKY53F	5′-GAAGTTGATGGAGGAGGAGAAG-3′
DoWRKY9F	5′-GTGATGGTATGGTGGGTCTATC-3′	DoWRKY53R	5′-CCTGGATGAAGTGTTGGTAGAT-3′
DoWRKY9R	5′-TGCTTCTTCATCCTCCAGTTC-3′	DoWRKY54F	5′-TGGGCAGAAGCCGATAAAG-3′
DoWRKY10F	5′-AGAGCCATGACTGCTAATTCC-3′	DoWRKY54R	5′-GGAGGAGTAGGTGACAATGAAC-3′
DoWRKY10R	5′-CTGCTCCTTCTCCTCATTCTTC-3′	DoWRKY55F	5′-GGCCACTTGATGACGGATATAG-3′
DoWRKY12F	5′-CGGTGAGGATGAAGTTGATAGG-3′	DoWRKY55R	5′-GCGGTATGTGCATCTGTAGTAG-3′
DoWRKY12R	5′-CCCTTCGTTCTCTCCTTCTTTC-3′	DoWRKY57F	5′-AGAACTGTAAGACGAGGAAGAAAG-3′
DoWRKY14F	5′-ATGACCAGGCGAAGAAGATG-3′	DoWRKY57R	5′-CCAACTAAGGCTCGGTAGTTATG-3′
DoWRKY14R	5′-CTCTTCGTCAGAAACGCAAATC-3′	DoWRKY59F	5′-CCGTCAGGGTGTTATATGAAGG-3′
DoWRKY15F	5′-ACAGAGCCGAGGATCATAGT-3′	DoWRKY59R	5′-TCATACTGGTTTGCTTGGATCA-3′
DoWRKY15R	5′-CCTTGGATGAGGGTTTCCTTTA-3′	DoWRKY60F	5′-AGGAACAGAAAGGTGGAGATG-3′
DoWRKY18F	5′-GCTACGCAAAGATGGAGGATAA-3′	DoWRKY60R	5′-CCGTACTTAGTCCACTCGTATTC-3′
DoWRKY18R	5′-GTGGCGGTTGAGATGAAAGA-3′	DoWRKY62F	5′-GTGGAGAAAGTATGGGCAGAAG-3′
DoWRKY20F	5′-TGGAAATGGAGGATCAGAATGG-3′	DoWRKY62R	5′-GTTGTAATCACCGCTCGTAAGT-3′
DoWRKY20R	5′-TCATATCGAGAGCCGCTTATTG-3′	DoWRKY63F	5′-GAGCTATCAGCAACCACCTTTA-3′
DoWRKY23F	5′-GAATAGGGTTGAGAGGAGCATAG-3′	DoWRKY63R	5′-CAGGCTTTGAGCCGAGATATT-3′
DoWRKY23R	5′-AATTTCCATGGCGGCAATAAG-3′	DoWRKY64F	5′-GCTCAGAAGACGGTGGTTATAG-3′
DoWRKY24F	5′-CTCGATCGAAGGCTGTAATGT-3′	DoWRKY64R	5′-GTACTTCCTCCATGACCAGTAATC-3′
DoWRKY24R	5′-CTGGTTGAGAGGATGGTGATTAT-3′	DoWRKY65F	5′-GAAGCTGCGAAAGAGAACAATC-3′
DoWRKY25F	5′-TCACGAACATGAAGGCAGAG-3′	DoWRKY65R	5′-CTTGTGAGATGGGAAGGAAGAG-3′
DoWRKY25R	5′-GAATCCTTTGGAGAACCGAGAG-3′	DoWRKY66F	5′-GCACTCACAGCAAAGACAAAG-3′
DoWRKY26F	5′-GGGTTTAGATGGAGGAAGTATGG-3′	DoWRKY66R	5′-GACCACGTACTTTGGAGGATTAT-3′
DoWRKY26R	5′-GAGCTGAGAGCACCTGTAATAG-3′	DoWRKY67F	5′-TGGCTTCTCCTAGCTCTCTATC-3′
DoWRKY27F	5′-AGCTACTACCGTTGCACAAATA-3′	DoWRKY67R	5′-CCTTCCTTCTAAGTTCCTGCTC-3′
DoWRKY27R	5′-GGCCTTCATAGGTTGTGATGA-3′	DoWRKY68F	5′-GGAAGGTAGAGGCTGAGTTTAAG-3′

DoWRKY28F	5′-AGCAAACTCATCTCAGCCTTTA-3′	DoWRKY68R	5′-TCTTTACAGCCTTCTTCCCATAC-3′
DoWRKY28R	5′-GCCGCCATTCTCCTCTATTT-3′	DoWRKY69F	5′-CCACATAACCTTGCCGATACA-3′
DoWRKY30F	5′-GTACGGGAAGAAGTCAGTCAAG-3′	DoWRKY69R	5′-GATGCGTTCCGCCGATAATA-3′
DoWRKY30R	5′-CGGGTCTTCGTTGTCTCTTT-3′	DoWRKY70F	5′-GGCGAGAGAATAATGGAGGATTG-3′
DoWRKY31F	5′-GGGAGATCACGATCATGCTAAG-3′	DoWRKY70R	5′-CAGCACCCGTTACCTGAATATC-3′
DoWRKY31R	5′-CCCTCCTTCCCAATCAAACA-3′	DoWRKY72F	5′-ACAAGGGATGCTGGAAGATG-3′
DoWRKY32F	5′-GGCTGTGAGTTTCCTTGTAGT-3′	DoWRKY72R	5′-TGAGATGAAGAGGGAGGAGTAG-3′
DoWRKY32R	5′-GTGGTTGTGAGAGCCCTTATAG-3′	DoWRKY73F	5′-CACTCCCTCGCTATCCATAAAC-3′
DoWRKY33F	5′-TGGCTTCTCCTAGCTCTCTATC-3′	DoWRKY73R	5′-CGCCATCTGCTCAATCATAAAC-3′
DoWRKY33R	5′-CCTTCCTTCTAAGTTCCTGCTC-3′	DoWRKY74F	5′-GGACGATCAGATCACGATTCAG-3′
DoWRKY35F	5′-GGAGGAAGAAGAGGAGGAAGAA-3′	DoWRKY74R	5′-GCAGACGATGAGGTGGATTT-3′
DoWRKY35R	5′-GCAACTTCTCCAAGCCCATAA-3′-3′	DoWRKY75F	5′-CATACCTGCAAGCCCATAGAG-3′
DoWRKY37F	5′-GTTGCTAGTGCCTCTCATTCT-3′	DoWRKY75R	5′-GCTGTAGGAAGAAGAAGCAGTAG-3′
DoWRKY37R	5′-CACTTGTTTCTGCCCGTATTTC-3′	DoWRKY76F	5′-GAACTGTAAGGCGAGGAAGAAG-3′
DoWRKY39F	5′-GACAAACCCACCTCCAGTTT-3′	DoWRKY76R	5′-GTGGTTTCTGAAGAGGCCATAG-3′
DoWRKY39R	5′-GCCACCTGTATCCATCACTTAC-3′	DoWRKY77F	5′-CTGATGGATTGGTTGTGAGAGA-3′
DoWRKY40F	5′-CGATAACGATGAACAAGCAAGAAG-3′	DoWRKY77R	5′-CTGGAGCAAAGGAGCATCTAA-3′
DoWRKY40R	5′-CTGAACCCTGGGAATCCATTAG-3′	DoWRKY78F	5′-CCAGAGAGCTCTGCATCATTAG-3′
DoWRKY42F	5′-ACTGGACTGCACCTTCTTTC-3′	DoWRKY78R	5′-TCTTCCTCGCTTTCTTGATTGA-3′
DoWRKY42R	5′-TTGCTGTTATCTCGCTCTTCTT-3′	DoWRKY79F	5′-AAGAGAGTGGAGAGAGATCGTAG-3′
DoWRKY43F	5′-TGGACCAACCAACTTCCTTATC-3′	DoWRKY79R	5′-CTTGGAGCTATGGCTAGTTGAG-3′
DoWRKY43R	5′-TGGACCAACCAACTTCCTTATC-3′	DoWRKY80F	5′-TTTCCACCAGGGATGGTTATG-3′
DoWRKY44F	5′-CCAGTTGAGGTTAGGGTTCTTT-3′	DoWRKY80R	5′-CAGCCACTACTTTAGGTGTTGA-3′
DoWRKY44R	5′-TTCATGCAGGGTTCCATCAG-3′	DoWRKY81F	5′-GGCAGCTCAAGCCCTAAAT-3′
DoWRKY45F	5′-GTGGGAGTGTTTCAGGGTTAG-3′	DoWRKY81R	5′-CACTCCCTTTCACTTGCTTCT-3′

Primer pairs for qRT-PCR were designed by online Primerquest software (http://www.idtdna.com/Primerquest/Home/Index).

Supplementary Table 2 The fold change of *DoWRKY* genes under cold stress in *D. officinale* root was calculated from the $2^{-\Delta\Delta CT}$ value.

Gene name	2 h		6 h		12 h	
	fold-change	regulation	fold-change	regulation	fold-change	regulation
DoWRKY1	2.265331	up	2.233163	up	4.533601	up
DoWRKY2	1.990573	up	3.328046	up	1.935483	up
DoWRKY3	0.979795		0.239189	down	0.542564	down
DoWRKY4	1.970462	up	1.91479	up	1.024765	
DoWRKY5	0.481705	down	0.240917	down	0.249185	down
DoWRKY6	2.330698	up	0.58959	down	0.567205	down
DoWRKY7	1.417782		1.329188		1.882226	up
DoWRKY9	1.039162		1.007853		1.038145	
DoWRKY10	1.853142	up	0.970247		1.958816	up
DoWRKY12	0.978066		0.945284		1.90043	up
DoWRKY14	0.977318		0.971165		1.909692	up
DoWRKY15	0.959762		0.956212		0.997179	
DoWRKY19	1.831244	up	0.972421		1.045971	
DoWRKY20	0.955991		1.911853	up	3.150004	up
DoWRKY23	0.99987		1.750872	up	1.002972	
DoWRKY24	1.082131		1.197936		1.02526	
DoWRKY25	1.009331		0.980022		0.965477	
DoWRKY26	1.036478		1.018927		0.508697	down
DoWRKY27	0.988178		0.967737		1.02529	
DoWRKY28	4.333651	up	4.066062	up	4.132963	up
DoWRKY30	1.059679		1.000311		1.030325	
DoWRKY31	0.941963		0.968469		0.498762	down
DoWRKY32	0.946468		0.934729		1.810887	up
DoWRKY33	0.516479	down	1.044661		0.139306	down
DoWRKY35	0.990622		0.937629		0.935037	
DoWRKY37	0.964434		1.906962	up	1.957952	up
DoWRKY39	1.838454	up	1.644565	up	1.733134	up
DoWRKY40	0.482648	down	0.763109		0.925449	
DoWRKY42	0.922696		0.94964		0.428906	down
DoWRKY43	1.047766		1.042897		1.139457	
DoWRKY44	1.071638		1.041361		0.55732	down
DoWRKY45	0.985298		0.970262		1.985393	up
DoWRKY47	0.977998		1.012522		1.01356	
DoWRKY48	1.021827		0.978902		0.498674	down
DoWRKY49	0.971073		0.959472		0.923272	
DoWRKY50	0.797415		0.617126	down	0.138434	down
DoWRKY51	0.939788		0.99726		2.092202	up
DoWRKY52	0.490264	down	0.972072		1.004103	
DoWRKY53	0.724382		0.204241	down	0.190358	down

DoWRKY54	0.958466		0.983483		0.970596	
DoWRKY55	1.006814		0.480759	down	0.485327	down
DoWRKY57	0.685186		0.364109	down	0.350887	down
DoWRKY59	0.976442		1.276076		1.798678	up
DoWRKY60	1.029446		0.943787		1.034011	
DoWRKY62	0.423143	down	0.403636	down	0.558543	down
DoWRKY63	1.013416		0.995385		0.526386	down
DoWRKY64	0.21684	down	2.015148	up	7.034233	up
DoWRKY65	4.020655	up	3.912917	up	7.969208	up
DoWRKY66	6.132598	up	1.174955		9.37194	up
DoWRKY67	2.434081	up	1.717512	up	1.83204	up
DoWRKY68	2.371172	up	0.908109		0.936863	
DoWRKY69	1.269489		1.241931		2.114103	up
DoWRKY70	0.824168		1.066812		1.417243	
DoWRKY72	0.827595		1.222205		0.854065	
DoWRKY73	0.836346		0.717944		0.293085	down
DoWRKY74	1.352469		0.844445		1.30155	
DoWRKY75	0.876421		0.899544		1.036603	
DoWRKY76	0.802102		1.011095		0.949802	
DoWRKY77	0.981828		1.073242		0.455477	down
DoWRKY78	0.932631		0.901152		0.48763	down
DoWRKY79	0.856348		0.626862	down	0.862714	
DoWRKY80	0.821365		0.936888		1.063025	
DoWRKY81	1.257885		1.101006		1.026375	

Supplementary Table 3 The fold change of *DoWRKY* genes under cold stress in *D. officinale* stem was calculated from the $2^{-\Delta\Delta CT}$ value.

Gene name	2 h		6 h		12 h	
	fold-change	regulation	fold-change	regulation	fold-change	regulation
DoWRKY1	1.978559	up	3.729832	up	3.727629	up
DoWRKY2	0.963284		0.958807		1.047581	
DoWRKY3	0.991002		0.840987		0.46765	down
DoWRKY4	0.515842	down	0.955601		0.961018	
DoWRKY5	0.989789		0.95229		0.489741	down
DoWRKY6	0.236484	down	0.311696	down	0.309769	down
DoWRKY7	0.499287	down	0.48959	down	0.497626	down
DoWRKY9	0.992045		0.942133		1.862031	up
DoWRKY10	0.505012	down	0.952971		0.980039	
DoWRKY12	1.029331		1.967985	up	1.963988	up
DoWRKY14	2.008905	up	1.978572	up	24.44417	up
DoWRKY15	0.982387		0.930468		0.919128	
DoWRKY19	1.020963		0.965149		0.978174	
DoWRKY20	0.996216		1.88666	up	1.768084	up
DoWRKY23	0.996287		1.119472		0.918883	
DoWRKY24	0.990862		0.957165		0.496485	down
DoWRKY25	1.015875		0.963873		0.975797	
DoWRKY26	1.933615	up	1.904293	up	0.902092	
DoWRKY27	0.498225	down	0.497844	down	0.479401	down
DoWRKY28	0.967644		1.928342	up	0.899999	
DoWRKY30	1.041043		0.965867		1.013071	
DoWRKY31	0.525282	down	0.764878		0.550235	down
DoWRKY32	1.064793		0.92683		0.932116	
DoWRKY33	0.736695		0.429154	down	0.293776	down
DoWRKY35	1.828691	up	1.840009	up	0.927246	
DoWRKY37	1.986425	up	1.968947	up	1.967264	up
DoWRKY39	1.017543		1.895791	up	1.894909	up
DoWRKY40	7.281612	up	11.0311	up	8.700599	up
DoWRKY42	3.995743	up	7.549858	up	7.064994	up
DoWRKY43	0.968988		1.158438		0.903429	
DoWRKY44	1.119839		1.139866		1.146968	
DoWRKY45	0.955679		0.926367		0.922151	
DoWRKY47	1.000923		0.977318		0.474632	down
DoWRKY48	0.987014		1.003871		0.463522	down
DoWRKY49	1.401035		1.645145	up	1.759266	up
DoWRKY50	0.577533	down	0.779937		0.757141	
DoWRKY51	1.068151		1.010333		1.010333	
DoWRKY52	0.511558	down	0.956512		0.974353	
DoWRKY53	0.31456	down	0.301076	down	0.174807	down

DoWRKY54	1.00862		0.482553	down	0.471822	down
DoWRKY55	1.97131	up	0.99776		0.953683	
DoWRKY57	0.902153		0.2787	down	0.222048	down
DoWRKY59	0.204969	down	0.159935	down	0.158516	down
DoWRKY60	3.093794	up	3.324029	up	1.270023	
DoWRKY62	3.219542	up	3.072899	up	1.33946	
DoWRKY63	1.051463		0.526551	down	0.51762	down
DoWRKY64	0.919817		0.787801		0.966855	
DoWRKY65	1.858142	up	3.24689	up	2.614288	up
DoWRKY66	1.198568		1.89081	up	4.722284	up
DoWRKY67	4.574224	up	4.080856	up	5.714229	up
DoWRKY68	1.078051		0.923888		1.139206	
DoWRKY69	2.219858	up	2.710736	up	3.966893	up
DoWRKY70	1.069007		1.098373		0.981706	
DoWRKY72	0.762916		0.719848		0.588838	down
DoWRKY73	0.877409		0.377186	down	0.560843	down
DoWRKY74	1.101376		0.983751		0.963808	
DoWRKY75	1.166543		2.184957	up	4.952336	up
DoWRKY76	0.830364		0.997346		0.92203	
DoWRKY77	0.877256		1.169656		0.958234	
DoWRKY78	0.97714		1.094654		1.158122	
DoWRKY79	0.912859		0.927852		0.996523	
DoWRKY80	0.8226		0.930583		1.017936	
DoWRKY81	0.917666		0.861284		0.936427	

Supplementary Table 4 The mean of FPKM value and fold change of *DoWRKY* genes under control and cold stress in *D. officinale* leaf.

gene name	control (FPKM mean)	cold treatment (FPKM mean)	fold change	regulation
DoWRKY1	37.58617	71.69538	1.907493634	up
DoWRKY2	15.4691833	32.09225	2.074592389	up
DoWRKY3	6.51223067	2.652049		
DoWRKY4	0.10295867	0.070751667		
DoWRKY5	13.352947	31.55624667	2.363242112	up
DoWRKY6	0	0		
DoWRKY7	27.5972333	13.59482	0.492615323	down
DoWRKY9	30.98927	27.15212667	0.876178325	
DoWRKY10	8.505595	5.690280333		
DoWRKY12	60.25857	18.59161	0.308530554	down
DoWRKY14	8.441295	29.08249667	3.445264816	up
DoWRKY15	34.8969567	47.93223667	1.373536298	
DoWRKY18	36.6834033	37.00455333	1.00875464	
DoWRKY20	15.8297733	17.197	1.086370578	
DoWRKY23	0.08892367	0.037388333		
DoWRKY24	2.29150933	0.4834		
DoWRKY25	57.2623067	77.15656333	1.34742325	
DoWRKY26	0.387865	0.227979333		
DoWRKY27	2.02340867	1.396272333		
DoWRKY28	0.12857933	0.018160333		
DoWRKY30	9.92869933	3.983945		
DoWRKY31	0.63641567	0.572590333		
DoWRKY32	2.098979	1.558955		
DoWRKY33	0.29632433	0.179211		
DoWRKY35	2.813717	3.708559		
DoWRKY37	9.139449	10.22312433	1.118571189	
DoWRKY39	16.0539933	16.12359	1.004335162	
DoWRKY40	4.783575	0		
DoWRKY42	38.2577567	48.31448333	1.262867652	
DoWRKY43	19.3435733	1.531395667	0.079168189	down
DoWRKY44	8.25805333	0.214149667		
DoWRKY45	56.3398633	25.22303333	0.447694258	down
DoWRKY47	0.77345367	0.312891		
DoWRKY48	0.12363667	0.127455667		
DoWRKY49	0.32230433	0		
DoWRKY50	0.35305167	0.094180333		
DoWRKY51	0.375303	0.124698667		
DoWRKY52	9.78057067	3.102335		
DoWRKY53	0.29487233	0.245218667		
DoWRKY54	10.8205053	6.922735333	0.639779301	down

DoWRKY55	13.2716433	1.382748	0.104188153	down
DoWRKY57	0.110151	0		
DoWRKY59	0.37069133	0.143092333		
DoWRKY60	0.37069133	0.143092333		
DoWRKY62	0.863337	0		
DoWRKY63	0.863337	0		
DoWRKY64	0	0.197575333		
DoWRKY65	28.56497	5.491928	0.192260941	down
DoWRKY66	1.62074967	0.191635		
DoWRKY67	0.29632433	0.179211		
DoWRKY68	0.03537267	0		
DoWRKY69	38.2577567	48.31448333	1.262867652	
DoWRKY70	0.16028533	0		
DoWRKY72	0.57739367	0.165394333		
DoWRKY73	5.22092933	1.015809667		
DoWRKY74	56.3398633	25.22303333	0.447694258	down
DoWRKY75	1.110346	0.895384		
DoWRKY76	0.01444067	0		
DoWRKY77	0	0.010516667		
DoWRKY78	62.96755	26.22064333	0.416415175	down
DoWRKY79	43.6600433	35.29937	0.808505153	
DoWRKY80	0.777636	0.453810333		
DoWRKY81	0	0		

Supplementary Table 5 The stress-related genes containing W-box elements in tier putative promoters.

Gene family	Annotation ID	W-box number	Position
Ethylene-responsive transcription factor	Dendrobium_GLEAN_10042967	3	-479, -431, -256
	Dendrobium_GLEAN_10049982	3	-698, -725, -892
	Dendrobium_GLEAN_10137828	3	-123, -194, -325
	Dendrobium_GLEAN_10056217	7	-253, -582, -505, -536, -511, -839, -11
	Dendrobium_GLEAN_10028885	3	-988, -140, -181
Disease resistance protein	Dendrobium_GLEAN_10110403	4	-551, -239, -589, -114
	Dendrobium_GLEAN_10136594	4	-438, -669, -399, -804
	Dendrobium_GLEAN_10028570	4	-675, -957, -591, -664
	Dendrobium_GLEAN_10064426	3	-390, -785, -117
Heavy metal transport / detoxification protein	Dendrobium_GLEAN_10020160	4	-963, -136, -98, -198
Peroxidase	Dendrobium_GLEAN_10006492	3	-730, -554, -169
	Dendrobium_GLEAN_10015395	3	-252, -150, -569
	Dendrobium_GLEAN_10021680	3	-153, -51, -437
	Dendrobium_GLEAN_10025405	6	-816, -628, -703, -9, -510, -124
	Dendrobium_GLEAN_10069749	4	-796, -96, -588, -913
NAC transcription factor	Dendrobium_GLEAN_10079456	3	-389, -558, -44
	Dendrobium_GLEAN_10034487	3	-344, -546, -34
	Dendrobium_GLEAN_10007482	3	-75, -65, -58
Dehydration-responsive element-binding protein	Dendrobium_GLEAN_10135906	3	-508, -25, -79
